# Sulfonated reduced graphene oxide catalyzed cyclization of hydrazides and carbon dioxide to 1,3,4-oxadiazoles under sonication

**DOI:** 10.1038/s41598-017-04143-4

**Published:** 2017-07-05

**Authors:** Manuri Brahmayya, Shenghong A. Dai, Shing-Yi Suen

**Affiliations:** Department of Chemical Engineering, National Chung Hsing University, 250 KuoKuang Raod, Taichung, Taiwan, R.O.C.

## Abstract

Acid catalysts facilitate many chemical reactions. Sulfonated reduced grapheneoxide (rGOPhSO_3_H) has shown to be an encouraging solid acid catalyst because of its efficiency, cost-effectiveness and safety of use. In this study, we prepared the rGOPhSO_3_H nano acid catalyst, with the introduction of aromatic sulfonic acid radicals onto GO by fractional removal of oxygenated functions. It was thoroughly characterized by FT-IR, X-ray diffraction (XRD), scanning electron microscopy (SEM), transmission electron microscopy (TEM), Raman spectroscopy, energy dispersive spectroscopy (EDS) and solid state ^13^C MAS NMR (SSNMR). Here we report the conversion of CO_2_ (1.0 atm pressure, at = 50 °C, the source of C_1_ carbon feed stock) with hydrazides and a catalytic amount rGOPhSO_3_H, which through a cyclization reaction results in a new strategy for the synthesis of 5-substituted-3H-[1,3,4]-oxadiazol-2-ones (SOxdOs) under ultrasonic irradiation. Hence this concept of cyclization opens up for new insights

## Introduction

**Figure 1:**
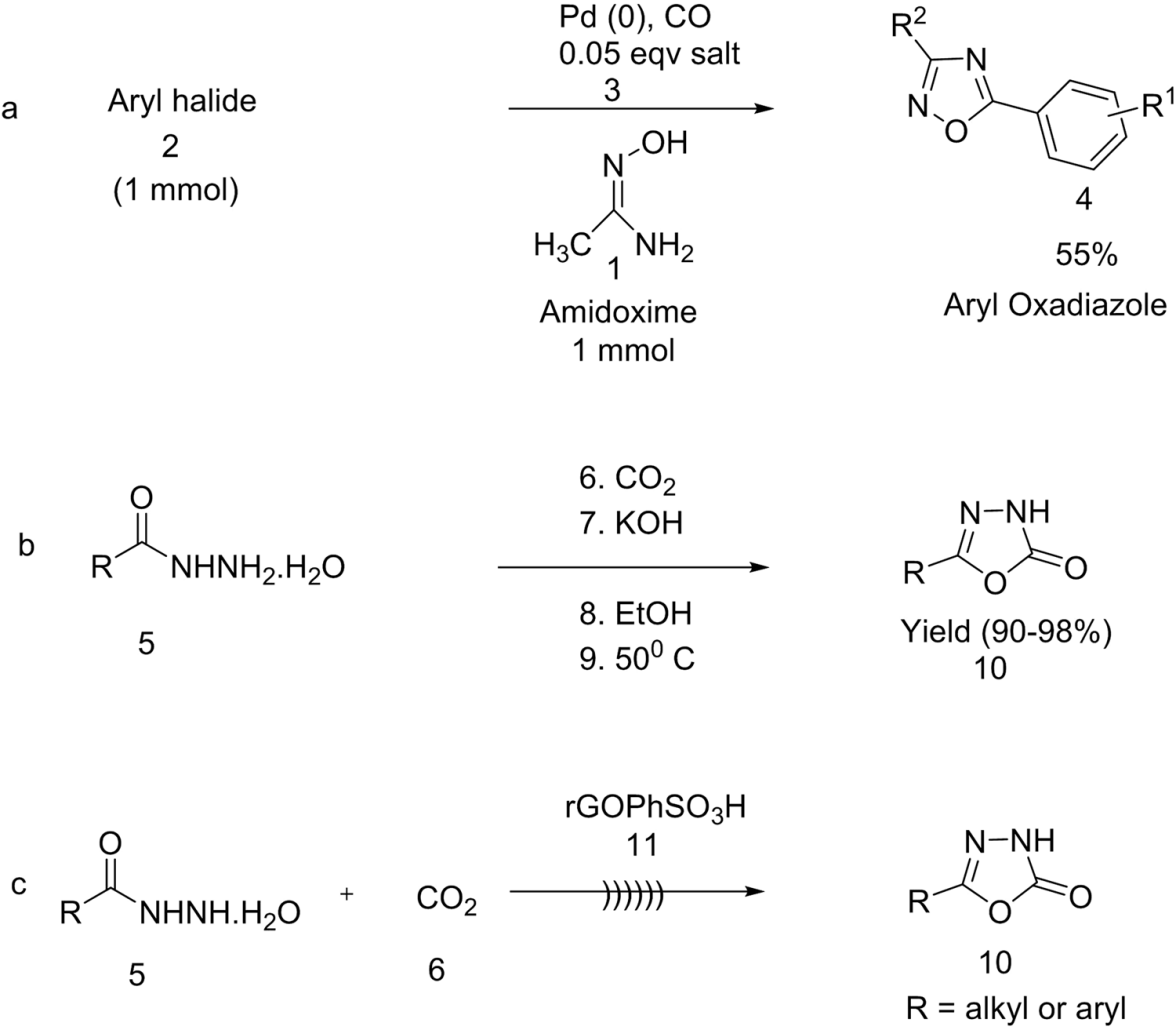
Previous developments of catalytic based 1,3,4-oxadiazloes with various substrates like amidoximes (**a**), hydrazides (**b**) and our current (**c**) synthetic applications respectively.

Synthesis of five membered 1,3,4-oxadiazoles or heterocyclic moieties elicits attention for their applications in active pharmaceutical ingredients, cell metabolism, drug intermediates, etc^1–5^.1,3,4-oxadiazoles are especially relevant due to their predictable activity and use as significant organic intermediates in the synthesis of antibacterial, antiviral, and other drugs^6–8^. The oxidative cyclization procedure constitutes one of the most favorable ways for preparation of these significant heterocyclic rings^9^. Several metal-based heterogeneous catalysts used in oxidative cyclization methods for 1,3,4-oxadiazoles synthesis results in the emission of the poisonous gas carbon monoxide (Fig. 1a)^10^. In other words, the insertion of carbonyl groups into organic molecules using oxidation or hydration is generally accomplished by using transition-metal catalysts which are often expensive, hard to eliminate, poisonous, and are obtained from inadequate natural sources^11^. Recently, we reported^6^ that carbon dioxide could effectively promote 1,3,4-oxadiazole cyclization process with hydrazides in the presence of a strong base and solvent (Fig. 1b). Still there were a few drawbacks in the reaction like, needing huge amount of solvent, strong basic medium and a long reaction times.

To seek a catalyst that covers the benefits of a metal-free and more efficient^6^ synthesis while retaining high catalytic activity, is an effort of serious importance in the preparation of 1,3,4-oxadiazoles^12–14^. Furthermore, solid acid catalysts have great potential to replace liquid acids, as environmentally friendly acid catalysts^15–17^. Acidic carbons, in view of green chemistry, were examined as stable and greatly active protonic acid catalysts for numerous acid catalyzed organic transformations^18, 19^. Graphene oxide, graphite oxide derivatives, and sulfonated carbon nanotubes have been meritoriously applied as advantageous heterogeneous catalysts for certain synthetic procedures^20–25^. Ultrasonic irradiation was applied to modify the graphite oxide and functionalized graphene oxide. Sulfonated graphene materials were recently reported for use in sonication nucleophilic substitution reactions^26–30^.

Herein, we report the use of sulfonated graphene oxide GOPhSO_3_H, a readily obtainable, efficient and mild carbon catalyst in ultrasonic pathways. More significantly, we investigated the first application of a sulfonated nano catalyst for the direct synthesis of 1,3,4-oxadiazoles from hydrazides with easily available, inexpensive, greenhouse gas and a C1 source, carbon dioxide (1.0 MPa).

**Figure 2:**
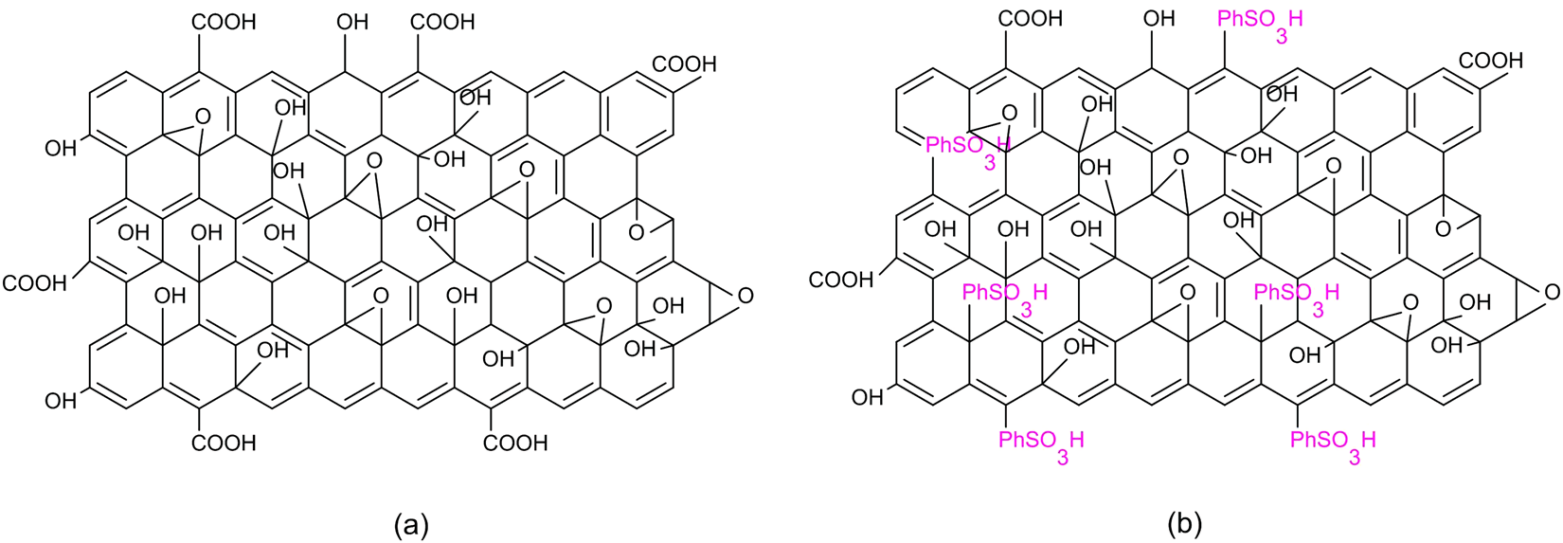
Model structures: (**a**) structural model of graphite oxide (GO) and (**b**) structural model of sulfonated reduced graphene oxide (rGOPhSO_3_H).

The history of graphite oxide (GO) reveals, it has functioned predominantly as a precursor to chemically enriched graphene material (CEGM) or reduced graphene oxide (r-GO), which have superior mechanical, chemical and electronic properties^31–33^. The synthesis of rGO^34, 35^ (Hummers method NaNO_3_ and KMnO4 in concentrated H_2_SO_4_) and GOPhSO_3_H^36–39^ usually involves the oxidation of graphite oxide. The harsh conditions used in these synthesis procedures introduce a diversity of oxygen-containing functionalities such as alcohols, epoxides, and carboxylates onto GO^34, 40^ and then conversion into GOPhSO_3_H (pH 4.8 at 0.1 mgmL^−1^)^37, 41^ material (Fig. 2).

A moderate catalyst has not been discovered for enabling the production of CEGMs^42, 43^. So far, nano carbon materials have been used to improve the performance exhibited by sustained transition metal-based catalysts, e.g GO that was intact with platinum nanoparticles used in hydrogen fuel cells and in methanol electro oxidation^44^. In the same way, GO imbued with palladium and silver nanoparticles exhibited very high turnover frequencies in some organic coupling reactions^45^.

## Results

**Figure 3:**
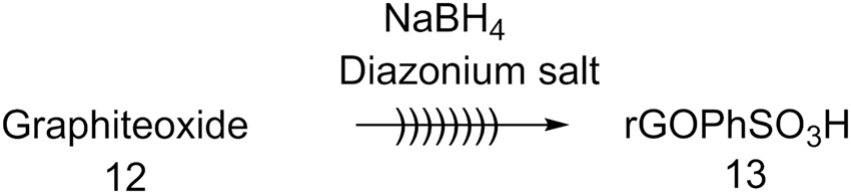
Synthesis of sulfonated reduced grapheneoxide.

**Figure 4:**
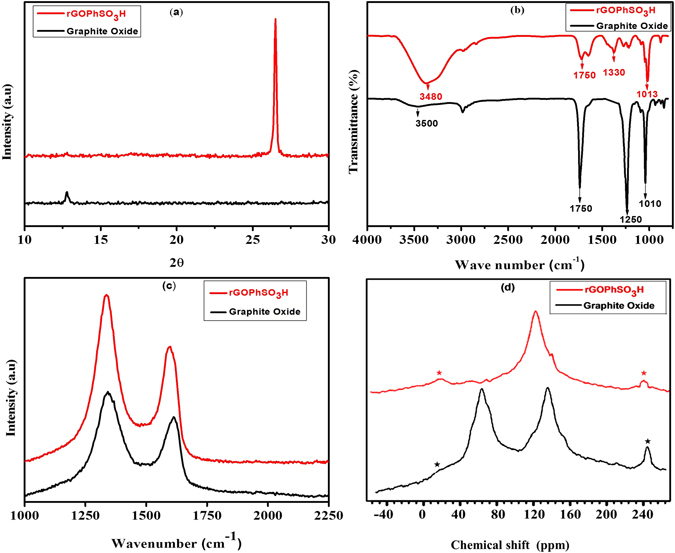
X-ray diffraction (**b**) FT-IR spectra, (**c**) Raman and (**d**) Solid state ^13^CMAS spectras of graphite oxide and sulfonated graphene oxide respectively.

Here, sulfonated graphene oxide was prepared by grafting sulfonic acid-containing aryl radicals onto reduced graphene oxide (rGO) (Fig. 3) under ultrasonic irradiations. The existence of countless chemical functionality on graphene oxide nano sheeted dispersion in water was studied by XRD, FTIR, Raman spectra, SEM, TEM, EDS and SSNMR. (Figure 4a) shows the XRD patterns achieved for both graphite oxide and sulfonated graphene oxide. The XRD pattern of graphite oxide exhibits a single diffraction peak at 2θ = 12.8° which confirms the formation of graphite upon the oxidation of graphite by the historical Hummer’s procedure^34, 37, 46^ (Fig. 4a). After the chemical reduction of exfoliated graphene oxide with sodium borohydride and subsequent grafting of sulfonic acid containing aromatic radicals, the usual peak of graphite oxide at 2θ = 12.8° (d = 6.91 Å) had been shifted to 2θ = 26.5° (d = 3.36 Å) for rGOPhSO_3_H^47^ (Fig. 4a), implying that the GO nano sheets were restacked via the л-л interaction upon sulfonation.

Further, the environment of the chemical functionalities over the rGOPhSO_3_H nanosheets were characterized by FTIR (Fig. 4b). A broad peak appeared at 3480 cm^−1^, attributed to the OH bond stretching mode, and depicts the abundance of increased hydroxyl groups due to SO_3_H in sulfonated graphite oxide. However, the hydroxyl groups in GO shows absorption at 3500 cm^−1^ with less intensity than rGOPhSO_3_H^37, 48^. A highly sharp and strong band at 1750 cm^−1^ (υ C=O) denotes the carbonyl group and carboxylic acid observed in both GO and the obtained rGOPhSO_3_H displaying the same band as highly decreased intensity, which signifies the partial reduction of graphite oxide in the sulfonation procedure. A significant peak appeared at 1330 cm^−1^ in rGOPhSO_3_H, which is due to the stretching of S=O bonds and confirms the successful sulfonation that occurred on the surface of the GO^48^. Furthermore, the sharp bands at 1250 cm^−1^ and 1010 cm^−1^ are ascribed to the presence of C–OH (hydroxy) and C–O (epoxy) groups, respectively in GO but the decreased band at 1013 cm^−1^ observed in rGOSO_3_H nanosheets, reveals successful sulfonation.

The Raman spectra of graphite oxide and sulfonated graphene oxide (Fig. 4c) provided a significant characterization in this study. Similar to the XRD and FTIR, these spectra provided evidence that there was ample presence of oxygen functionalities, such as hydroxyl, epoxide, carboxylate and carbonyl on graphite oxide, shown in (Fig. 4c). A couple of characteristic peaks at 1310 and 1593 cm^−1^ for graphite oxide and at 1312 and 1584 cm^−1^ for rGOPhSO_3_H occur in each testing spectra, which corresponds to the D and G bands, respectively^49^. The I_D_/I_G_ ratio in rGOPhSO_3_H (2.06) > graphite oxide (1.54). This I_D_/I_G_ ratio difference suggests that some of the oxygenated functions are detached by NaBH_4_ under the applied sonication method^37, 45–52^.

**Figure 5:**
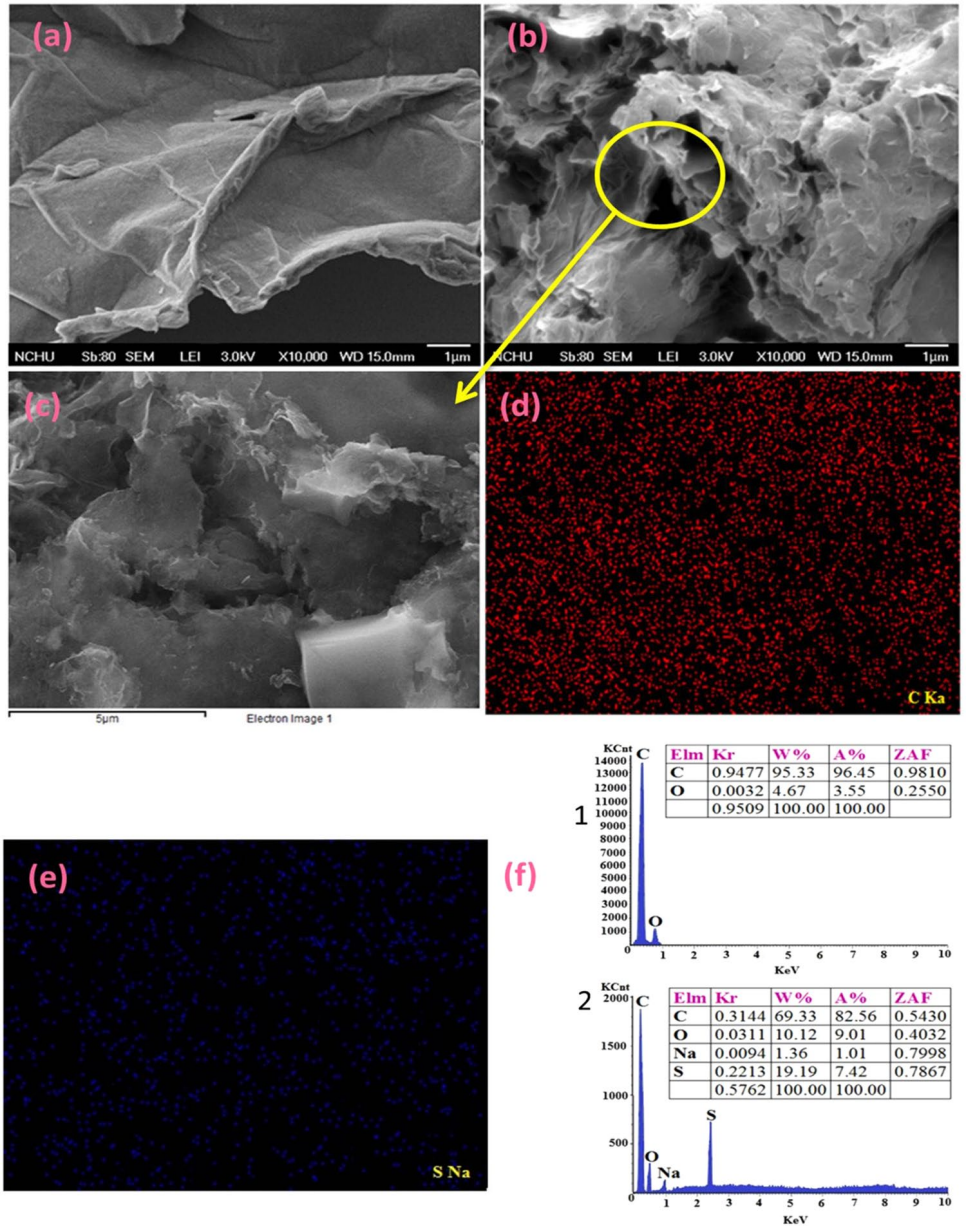
SEM images of (**a**) Graphite oxide, (**b**) synthesized sulfonated graphene oxide, (**c**) shows the SEM image of a huge area of dispersed powder of rGOPhSO_3_H on the silicon wafer for elemental analysis. (**d**) and (**e**) denote the resultant EDS elemental mapping of C and S of the region which shown in (**e**). (f 1 and f 2) displays the EDS elemental analysis of graphite oxide and sulfonated graphene oxide.

**Figure 6:**
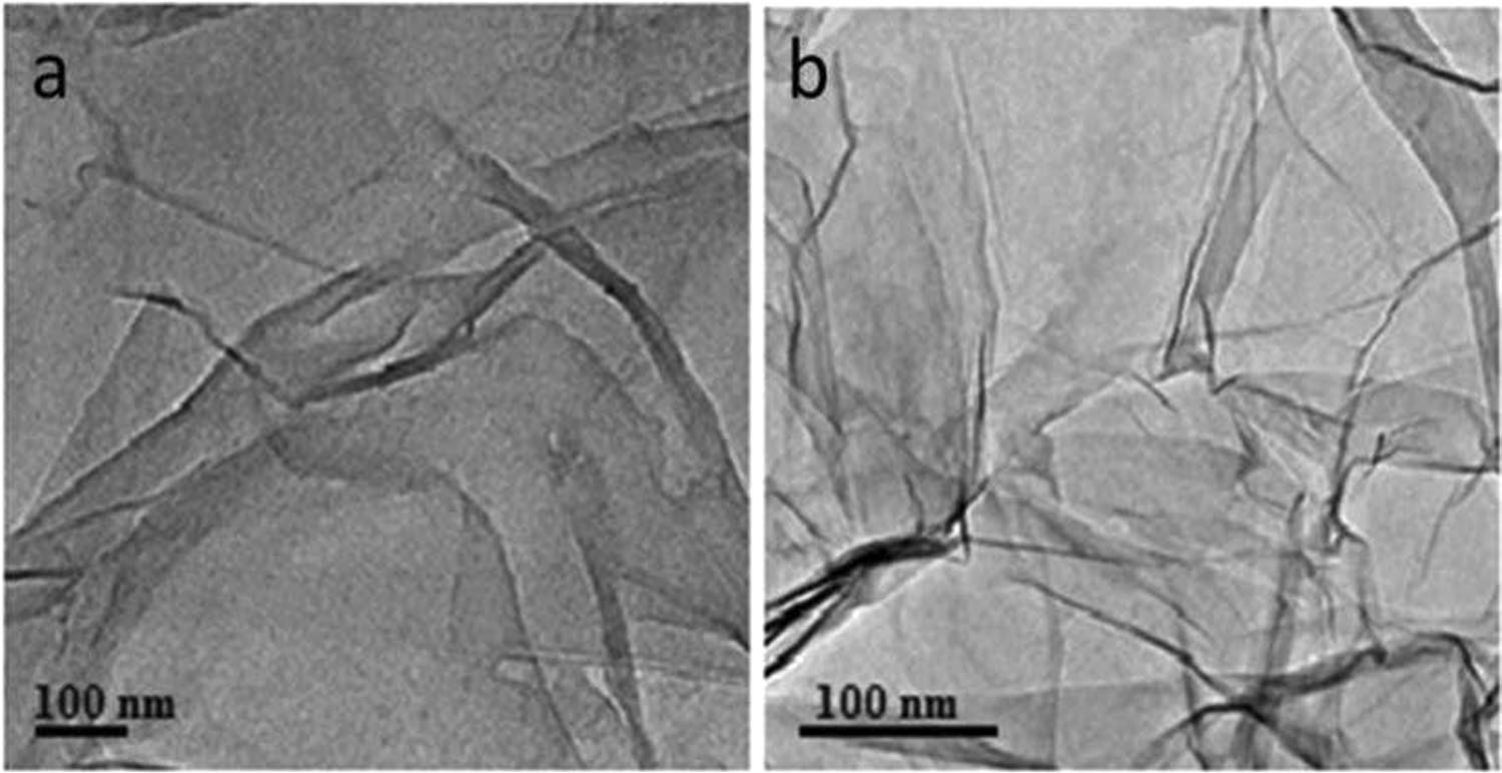
TEM photographs (**a**) graphite oxide (**b**) sulfonated graphene oxide.

Furthermore, the presence of morphological characteristics on the SEM images^53^ (Fig. 5a and b) of graphite oxide and rGOPhSO_3_H nanosheets confirm the successful exfoliation after embedding sulfonic acid aryl radicals onto modified rGO under ultrasonic irradiation. The EDS elemental mapping of carbon (Fig. 5d) and sulfur (Fig. 5e) in a large area (Fig. 5c) of GOPhSO_3_H dispersed powder demonstrates a homogeneous distribution of PhSO_3_H functionalities on the graphite oxide structure^53^. The EDS elemental analysis (Fig. 5f1 and 5f2), reveals 69.33, 10.12, 19.19 weight% (W%) and 82.56, 9.01, 7.42 atomic % (A%) for C, O and S, respectively. Based on our sources, such high sulfur content in graphite oxide structure has not been test before. Transmission electron microscopy (TEM) confirmed^53^ that the microstructure of the graphite oxide sheets was not damaged by the sulfonation reaction (Fig. 6). This wrinkle property may have possible benefits in acid catalyzed reactions, as the substrates can effortlessly access the active sites on both side surfaces of the 2D graphene nano sheets. Nevertheless, the 2D molecular structure may be the reason for the diffusion hindrance in the reaction, the reduced sulfonated graphene nano sheets sufficiently small enough (5 μm × 1 nm in size) to be well propagated or dispersed under stirring conditions. The wrinkling facet with scarce micropores may assist the diffusion of the desired products.

Solid state ^13^C MAS NMR (SS^13^C NMR) also endorse the graphite oxide reduction (Fig. 4d). A couple of dissimilar resonances aroused in the spectrum of graphite oxide: the resonance centered at 132 ppm represents to an oxidized sp^2^ carbons. Resonance centered at 64 ppm corresponds to epoxidation, and the 72 ppm shoulder is derived from hydroxylated carbons^31, 54, 55^. The later weak broad resonances overlap at 158 corresponding to carbonyl carbons. The resonance peak at 132 ppm shifts to 118 ppm in rGOPhSO_3_H due to the change in the chemical environs of the sp^2^ carbons^54^. The resonances at 72 ppm and 158 ppm disappear with a significant small peak roused at 142 ppm is attributed to carbons in the chemically (covalently) attached phenyl-SO_3_H groups.

## Discussion

### Substrate scope

**Figure 7:**
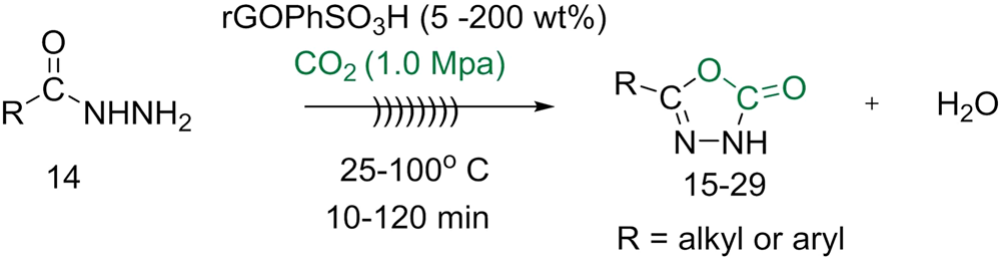
Synthesis of 5-susbstituted-1,3,4-oxadiazole-2-ones.

The catalytic potential of sulfonated graphene oxide nanosheets was examined for the cyclization of hydrazides to 1, 3, 4-oxadiazoles for the first time as shown in (Fig. 7).

In a typical experimental procedure, we examined the direct cyclization of 4-methyl benzoyl hydrazide with CO_2_ under different conditions. As summarized in Table 1 (See Table S1 for the additional optimization studies) the cyclization properties of the reaction are as follows: With a neat benzohydrazide (1 mmol) and CO_2_ (1.0 MPa), no reaction was observed in the absence of nano catalyst at room temperatures (RT) or 50 °C. The reaction also failed to afford the 1, 3, 4-oxadiazole moiety with the use of natural flake graphite oxide (20 wt %) at RT (48 h) and 50 °C (24 h) in conventional reaction procedure. At temperature 50 °C, in presence of synthesized nano catalyst rGOPhSO_3_H the percentage conversion is at 20%, after the reaction period 24 h. Inspired by this, we tried the reaction with reproduced reduced graphene oxide^34, 37, 40^ (rGO, 20 wt%, 50 °C, 24 h) to determine its fate in cyclization procedure but unfortunately no reaction was observed. Inspired by the improved reaction yield 20% from the nano catalyst rGOPhSO_3_H conventional reaction condition, we then screened our reaction possibilities under sonication. At room temperature, the percentage of the conversion hiked at 70% (10 h, with catalyst loading 5 wt %) whereas the reaction with nano catalyst (5 wt %) at 50 °C yields 84% POxdO in just 50 min (under ultrasonication). Unfortunately, the reaction yield was reduced from 84% to 76% with the same catalyst quantity at 70 °C. Maintaining the constant time factor 50 min, with various catalyst loadings (20 wt%, 50 wt%, 200 wt %) and temperatures (50 °C, 70 °C) under applied sonication bath, the best conversion of 5-phenyl-3H-[1,3,4]-Oxadiazol-2-one (POxdO) afforded (99%) at 50 ^°^C with 200 wt% for the hydrazide vs carbon dioxide (1 mmol: *P*_CO2_ = 1.0 atm) rather than higher temperature 70 °C. The results summarized in Tables 1 and S1 reveals that the reaction was initiated backward for more than 50 °C with an assisted ultrasonic irradiation. This indicates that the reaction should follow the determined reaction conditions only.

**Table 1:** Investigation of several reaction conditions for the cyclization of 4-methyl benzohydrazide with carbondioxide into 5-phenyl-3H-[1, 3, 4]-oxadiazol-2-one (PoxdO)^a^.

Catalyst	Loading [Wt%]	T (^°^C)	Time (Min)	Ultrasonic bath	PoxdO (Yield %)
—	—	RT^b^	48 h	—	0
—	—	50 °C	48	—	0
Graphite oxide	20	RT^ c^	48 h	—	8
Graphite oxide	20	50^d^	24 h	—	6
GOPhSO_3_H	20	50^e^	24 h	—	20
rGO	20	50	24 h	—	0
GOPhSO_3_H	5	RT	10 h	Applied	70
GOPhSO_3_H	5	50	50 min	Applied	84
GOPhSO_3_H	5	70	50 min	Applied	76
GOPhSO_3_H	20	RT	50 min	Applied	60
GOPhSO_3_H	20	50	50 min	Applied	83
GOPhSO_3_H	20	70	50 min	Applied	76
GOPhSO_3_H	50	RT	50 min	Applied	67
GOPhSO_3_H	50	50	50 min	Applied	88
GOPhSO_3_H	50	70	50 min	Applied	80
GOPhSO_3_H	200	RT	50 min	Applied	69
GOPhSO_3_H	200	50^f^	50 min	Applied	99
GOPhSO_3_H	200	70	50 min	Applied	70

(See Table S1 for further optimization studies).

^a^Reaction conditions: 4-methyl benzo hydrazide (1 mmol), carbon dioxide (1 atmospheric pressure), GOPhSO_3_H (200 wt%). ^b^blank experiment at room temperature without graphite materials and ultrasonic bath, ^c,d^Trial experiments with graphite oxide at RT and 50 °C respectively in the absence of ultrasonic irradiation, ^e^Experiment carried with sulfonated graphite oxide, under ultrasonic bath, ^f^Successful reaction carried out using GOPhSO_3_H under ultrasonication.

Next, we expanded the possibility of the reaction by using aliphatic and aromatic hydrazides under the determined reaction conditions (Table 1) to produce the corresponding cyclized 1,3,4-oxadiazoles (Table 2). The obtained results are displayed in Table 2 (entries 15–29). All of the substrates were efficiently cyclized to the corresponding 5-substituted-3H-[1, 3, 4]-oxadiazol-2-ones [SOxdOs] in tremendous yields within 50 min without generation of other waste products. Among the various hydrazides, acetohydrazide is reinforced and accelerated by + I effect^6^ and converted to 5-methyl-3H-[1,3,4]-oxadiazol-2-one (Entry 15, Table 2) i.e the flow of electrons towards the third most electronegative element nitrogen from methyl in acetohydrazide boosted up the reaction within stipulated time of sonication. As in conventional aspect, aromatic substrates with electron donating groups are readily assisting the cyclization to achieve the titled compounds (Table 2, entries 19, 21, 25, 27, 28). In the case of aromatic hydrazides that contain electrons with drawing groups, these cyclized into relatively lower yields (Table 2, entries 16, 17, 18, 20, 22, 23, 24, 26, 29) compared with their relative substrates^3, 4^. In particular, it is noteworthy to mention that thiophenol and phenol were tolerated in the reaction without need for any protecting groups in synthetically useful yields at 96% (Table 2, entries 22, 24). Thus the reaction achieved satisfactorily useful yields, even though it is slightly sensitive to substrate electronic effects. All of the obtained products were assessed by HRMS (ESI), IR & ^1^HNMR, ^13^CNMR.

**Table 2:** Ultrasound-assisted direct cyclization of various hydrazides to 5-substituted-3H-[1, 3, 4]-oxadiazole-2-ones (SOxdOs) using rGOPhSO_3_H nano catalyst.

Entry	Hydrazide	5-substituted-1,3,4-oxadiazole-2-ones	Yield (%)^a^
15			91
16			88
17			90
18			91
19			98
20			89
21			99
22			96
23			92
24			96
25			99
26			87
27			98
28			94
29			86

^a^Isolated product.

### Recyclability of the catalyst

Additionally, we studied the recycling of the rGOPhSO_3_H catalyst by selecting the reaction of 4-methoxy benzoyl hydrazide and carbon dioxide as a model reaction. Once the reaction was completed, the catalyst was easily recovered by extraction of the reaction mass with dichloromethane. The resultant aqueous layer holding catalyst was used for the subsequent experiments totaling 7 runs with the same catalyst. These experimental results are shown in Table 3. As shown, reaction time and the yield of the product was considerably found to be alike throughout the 4 reaction cycles, establishing the effective recycling of the catalyst, as well as the heterogeneous catalytic property of the established method. Yes, the reaction cycles 5–7 are evidently decreasing, which reveals that elsewhere the leaching of SO_3_H function is very common problem for the sulfonated acid catalysts^39, 56^. Further advances should be made in the near future to increase the stability of the SO_3_H function. It is noteworthy to explore the chemical causes for the decreased yields in the reaction cycles from 1 to 7. The leaching of SO_3_H during the recycling reactions and thus the deactivation of rGOPhSO_3_H may be caused by (i) decrease in the quantity of catalyst during the recycling process and (ii) the possibility of the free radical (SO_3_H) substitution with substrate or mostly with hydroxy radicals under the ultrasonication^57^.

**Table 3:** Recycling experiments^a^.

Run	1	2	3	4	5	6	7
Time (Min)	50	50	50	50	50	50	50
Yield (%)^b^	91	90	89	86	60 (90)^c^	53 (89)^d^	50(89)^e^

^a^Conditions as mentioned in the text, ^b,c,d,e^The number in bracket specifies the yield of 1,3,4-oxadiazole by using the recycled acidified rGOPhSO_3_H catalyst.

**Figure 8:**
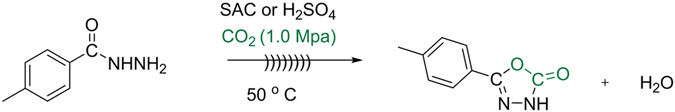
Synthesis of compound 27 either by using SAC or H_2_SO_4_.

On the other hand, the catalytic activity of rGOPhSO_3_H was also compared with the H_2_SO_4_ and sulfonated active carbon (SAC) (Fig. 8). The resulting yields of compound 27 via the rGOPhSO_3_H, SAC, and H_2_SO_4_, are 98, 45 and 38% respectively. This shows that the catalytic activity of rGOPhSO_3_H is higher than with its colleague catalysts. Distinguished yields also suggest that the catalytic activity for the cyclization of substrates is not solely dependent on the catalytic acid densities but the reaction might be forwarded by the following literature reports^58, 59^. (i) accessibility of the active sites, (ii) including weekly acidic OH, COOH groups (e.g Bronsted acid sites) alongside the strongly acidic SO_3_H on GO, (iii) the specific surface area of the nano catalyst and (iv) pore diameters of the prepared nano catalyst.

### Influence of the sonication on the reaction

For mechanical special effects, cavitation prompted by ultrasound can support several heterogeneous and homogeneous reactions. The main principle of many claims of ultrasound is its acoustic cavitation. The generation of tiny bubbles greatly produces high pressure and heat. Key step of this application is to enhance the temperature and pressure of upto various thousand degrees (Kelvins). It is anticipated that the generated cavitation bubble delivers the needful energy for the reaction. The reaction mechanism for direct cyclization of hydrazides with carbon dioxide using sulfonated graphene oxide catalyst under ultrasound irradiation is not very clear at this stage. Still, we proposed a reasonably plausible mechanism with our best of acquired knowledge in sonochemistry^60^.

**Figure 9:**
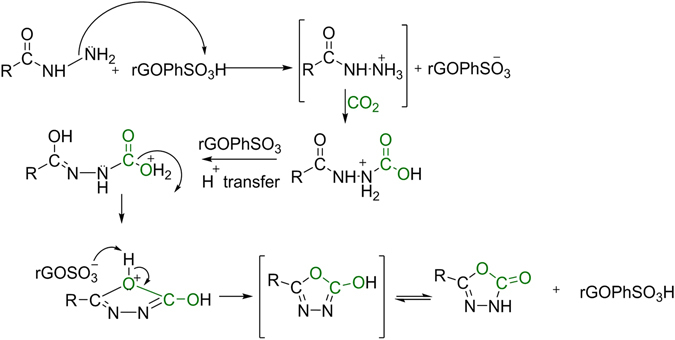
Postulated reaction mechanism of 5-substituted-3H-[1,3,4]-Oxadiazol-2-ones (SOxdOs) under ultrasonication.

It is noteworthy to mention that the presence of phenyl sulfonated functionalities on the surface of GO plays a significant role in the activation of substrates and carbon dioxide to produce targeted oxadiazoles. The outlined mechanism is shown in (Fig. 9). The reaction initially forms of a stable hydrazide ammonium salt. This salt would be gone through a subsequent addition of carbon dioxide to release N-substituted carboxylic acid and then N-substituted carboxylate. It was anticipated that the influence of ultrasonication, higher acidic capacity of rGOPhSO_3_H catalyst and the increased generation of the N-Substituted carboxylate salt would force a water elimination step thereby including the 5-membered cyclization with protonated oxygen. Eventually, removal of hydrogen from the oxygen of 1, 3, 4-oxadiazole moiety to GOPhSO_3_^−^ anion to recycle the catalyst.

## Conclusion

In summary, a simple procedure was used to prepare rGOPhSO_3_H nanomaterial by grafting sulfonated phenyl radicals onto the surface of rGO under ultrasound irradiation. GOPhSO_3_H/CO_2_ was the tested system used to catalyze the cyclization involving oxidation of various hydrazides under sonochemical conditions. These results were accomplished to under mild conditions and produced 5-substituted-3H-[1, 3, 4]-oxadiazol-2-ones in outstanding yields. Additionally, recovery of catalyst was found to be both effective and convenient with use of very simple filtration procedures. These results establish the very first time a methodology to achieve 1,3,4-oxadiazoles uses a metal free carbocatalyst to support synthetically useful transformations. In a wider scope, the potentiality of rGOPhSO_3_H or other 2D arrays of carbon materials now covers beyond the application of their significant electronic and mechanical properties. Further developments of the reaction are under investigation.

## Materials and Methods

All chemical were purchased from the local commercial sources and were used without further purification. Unless otherwise distinguished, all experiments were achieved with handy reaction conditions. ^1^H and ^13^C NMR data were collected on a 400 MHz spectrometer and a 100 MHz spectrometer respectively. Chemical shift (δ) values are referenced downfield from tetramethylsilane using the residual solvent as an internal standard (All synthesized compounds were dissolved in DMSO-d6 to analyze in NMR instruments). The mass spectra were performed by a Finnigan LTQ-Orbitrap XL instrument (ESI source). Fourier transform Infra-Red spectra were recorded with a Perkin-Elmer Spectrum One FT-IR spectrometer. X-ray diffraction was performed on MacScience MXP3 (λCu Kα = 0.154 nm) operated at 40 kV and 30 mA. The collection interval and scanning rate were 0.02° (θ/2θ mode) and 5°/min, respectively. Micro-Raman scattering experiments were achieved using a Dilor X–Y 800 spectrometer (spectral resolution of 0.6 cm^−1^ at room temperature in air). An argon-ion laser with wavelength of 514.5 nm and with the power of 8 mW, as well as the diameter of 1 μm was used for the excitation. The spectra were performed over the wavenumber from 200 to 800 cm^−1^ with an intervaled time of 600 s, and the spectra were then deconvoluted for defining the appropriate peak position. The entire spectra were standardized to the He–Ne laser excitation line. Scanning electron microscope (SEM) a model JEOL-JSM6700F instrument (Japan) was used for the morphology studies and EDX spectra. For high resolution transmission electron microscope studies, HR-TEM, model JEM-2010, JEOL, Tokyo, Japan was used. 4mm CP/MAS DR probe solid state ^13^CNMR MAS resonance spin rate at 10 KHz was used.

### Preparation of sulfonated grapheneoxide (GOPhSO_3_H)

The rGOPhSO_3_H nano catalyst was synthesized accordingly to a known literature^37–39^ procedure with minor modifications. Initially, 100 mg of graphite oxide powder was added into a glass flask with 100 ml of deionized water for 10 minutes and mixed uniformly with a magnetic stirrer at room temperature. The resulting dispersion was transferred to a vessel, and then sonicated in a ultrasonic bath (20KHz with 100% output). A solution of 0.8 g NaBH_4_ in 20 mL of deionized water was slowly added dropwise into the graphene oxide dispersion, with adjusting pH of 10 by addition of 5 wt % potassium carbonate solution. Then the dispersion was heated up at 100 °C for 45 minutes during which the brown colored GO dispersion turned out as black. Later, the reaction mixture was washed with deionized water and centrifuged. The obtained incompletely reduced graphene oxide (rGO) was irradiated in 100 mL water using ultrasonic bath with a frequency of 20 kHz for 30 min and cooled. Afterwards, a diazonium salt was synthesized from the reaction of 100 mg sulfanilicacid and 1.1 mL of 1 N HCl in 15 mL water and cooled at 0 °C, monitored by adding 35 mg KNO_2_ in 15 mL water. Thereafter, diazonium solution had been poured into the obtained rGO solution at 0 °C and stirred overnight 25 °C. The resulting black precipitate solution was then centrifuged at 7000 rpm for 10 min and washed five times with deionized water. The subsequent black residue was filtered under vacuum and dried in an oven at 70 °C for 4 h.

### Synthetic procedure for the preparation of sulfonated active carbon

With petty modification of the previous report^61^, the activated-carbon (powder, 1.0 g) was taken into concentrated H_2_SO_4_ (18 mol L^−1^, 25 mL) and heated under inert atmosphere 150 °C for 10 h. The obtained SAC was washed continuously (at least 6 times) with hot deionized water (2.5 L) and then with acetone, and later it was dried at 80 °C. Moreover, the achieved black powder was pre-treated hydrothermally at 200 °C for 2 h and then washed further with hot deionized water until the sulfate ions are no more eluted and detect in the washed water. Further, the density of sulfonated groups (SO_3_H) on the active carbon catalyst was determined by the back acid–base titration as 1.5 mmol g^−1^.

### General procedure for the synthesis of 5-(4-methyl phenyl)-3H-[1, 3, 4]-oxadiazol-2-one by using heterogeneous sulfonated active carbon (SAC)

The catalytic activities of the sulfonated active carbon were studied through the cyclization of 4-methyl benzohydrazide (MBH) with CO_2_ at 50 °C. Typically, MBH (1 mmol), CO_2_ (1.0 atm.pressure, at = 50 °C), and the sulfonated active carbon (SAC) catalyst (0.2 g, 1.5 mmol g^−1^ = SO_3_H) were utilized in the reaction. The reaction mass was later checked by thin layer chromatography (TLC). The achieved reaction crude was cooled to the room temperature. Then, the catalyst was separated out via the filtration and it was washed with ethanol. The obtained filtrates were subjected to evaporate under the reduced pressure to afford a residue. Thus to isolate the compound 27 (45%), afterwards it was recrystallized in EtOH.

### General procedure for the synthesis of 5-(4-methyl phenyl)-3H-[1, 3, 4]-oxadiazol-2-one by using homogeneous H_2_SO_4_

In a 50 mL round-bottomed flask, a mixture of homogeneous H_2_SO_4_ (0.146 g, ρ (density) = 1.48 mmol g^−1^), 4-methyl benzohydrazide (1 mmol) with CO_2_ (1 atm.Pressure) was heated at 50 °C under ultrasonication and stirred until TLC shows the completion of the reaction. Thus, the achieved reaction mixture cooled to room temperature and extracted with ethyl acetate (2 × 5 ml) by using separating funnel. Organic layer and aqueous layers were separated. The extracted organic layer was subjected to dehydration over Na_2_SO4, filtered and vaporized under reduced pressure. Purification of the obtained sample was accomplished by column chromatography using n-hexane/EtOAc: (9:1) as an eluent to afford 38% of 5-(4-methyl phenyl)-3H-[1, 3, 4]-oxadiazol-2-one (27).

### Typical procedure for the hydrazides with 1 atm.CO_2_ catalyzed by rGOPhSO_3_H under ultrasonication

4-methyl benzohydrazide (1 mmol) was dissolved in ethanol (5 mL) in a 25 mL three necked round bottom (RBF) flask under ultrasonic bath with a frequency of 20 kHz and 100% output power) with a magnetic stirrer inside to mix the reaction. The reaction mixture was well stirred. Then, GOPhSO_3_H (Carbocatalyst) (5–200 wt%) was added into the reaction mixture and stirred for 10 minutes to attain a homogeneous reaction mass in the flask at room temperature (RT). Carbon dioxide (1atm) was passed into the reactor via the other available neck of the RBF with the help of the gas purging glass tube. Slowly the temperature was raised to 50 °C and was continued for 30 min. To monitor the extent of cyclization, aliquots were removed at various time intervals, mixed with DMSO-d_6_ (1 mL), filtered off, to remove GOPhSO_3_H, and then analyzed by NMR spectroscopy. Later, the CO_2_ inlet was detached and the reactor neck was sealed and the reaction mass was refluxed for 10 minutes to achieve a complete crude product. This crude product was then quenched with dilute HCl (5%) and then 5 mL of ethyl acetate was added. The mixture was then filtered through a sintered funnel and again extracted with an ethyl acetate. The organic layer was then dehydrated over Na_2_SO4, filtered and vaporized under reduced pressure. Purification of samples was accomplished by column chromatography using n-hexane/EtOAc: (9:1) as an eluent. For NMR data charts of all compounds (compounds **15**–**29**), see the Supplementary Information S2–S16.

### Spectral Data for compounds 15–29

**5**-**methyl**-**3H**-[**1**, **3**, **4**]-**oxadiazol**-**2**-**one** (**15**). IR (KBr) νmax: 2985.13, 1754.52, 1512.85, 1150.14, 1030.47 cm^−1^. ^1^HNMR (400 MHz, DMSO-d6) δ 10.20 (brs, 1H,CONH), 2.00 (s, 3 H, CH_3_). ^13^CNMR (100 MHz, DMSO-d6) 154.11, 153.63, 18.30. HRMS (ESI) calcd for [C_3_H_4_N_2_O_2_]^+^: 101.07. Found: 101.04.

**5**-(**4**-**trifluoro phenyl**)-**3H**-[**1**, **3**, **4**]-**oxadiazol**-**2**-**one** (**16**). IR (KBr) νmax: 3010.39, 2976.43, 1760.00, 1489.85, 1150.14, 1100, 1011.33 cm^−1^. ^1^HNMR (400 MHz, DMSO-d6) δ 10.47 (brs, 1H, CONH), 7.63 (dd, J = 8.0, 2H, ArH) 7.0 (dd, J = 7.4Hz, 2H, ArH). ^13^CNMR (100 MHz, DMSO-d6) 155.55, 154.34, 129.80, 129.00, 125.00, 124.35, 121.36. HRMS (ESI) calcd for [C_9_H_6_F_3_N_2_O_2_]^+^: 231.06. Found: 231.01.

**5**-(**4**-**chloro phenyl**)-**3H**-[**1**, **3**, **4**]-**oxadiazol**-**2**-**one** (**17**). IR (KBr) νmax: 3020.17, 2852.33, 1755.10, 1480.85, 1135.12, 1100, 1016.11 cm^−1^. ^1^HNMR (400 MHz, DMSO-d6) δ 10.40 (brs, 1H, CONH), 7.66–7.80 (m, 4H, ArH). ^13^CNMR (100 MHz, DMSO-d6) 150.36, 155.00, 154.78, 138.00, 129.01, 128.89, 128.33, 127.99, 127.32. HRMS (ESI) calcd for [C_8_H_6_ClN_2_O_2_]^+^: 197.03. Found: 197.00.

**5**-(**4**-**bromo phenyl**)-**3H**-[**1**, **3**, **4**]-**oxadiazol**-**2**-**one** (**18**). IR (KBr) νmax: 3010.10, 2890.20, 1753.01, 1469.85, 1140.15, 1090, 1020.20 cm^−1^. ^1^HNMR (400 MHz, DMSO-d6) δ 10.00 (brs, 1H, CONH), 7.64–7.80 (m, 4H, ArH). ^13^CNMR (100 MHz, DMSO-d6) 161.00, 160.01, 138.89, 124.98, 124.00, 123.69, 123.22, 122.22. HRMS (ESI) calcd for [C_8_H_6_BrN_2_O_2_]^+^: 240.03. Found: 240.10.

**5**-(**2**-**methoxy phenyl**)-**3H**-[**1**, **3**, **4**]-**oxadiazol**-**2**-**one** (**19**). IR (KBr) νmax: 3001.10, 2900.23, 1750.23, 1480.89, 1158.12, 1087, 1019.01 cm^−1^. ^1^HNMR (400 MHz, DMSO-d6) δ 10.00 (brs, 1H, CONH), 8.2 (dd, 1H, J = 7.5 Hz, ArH), 7.8 (dd, 1H, J = 7.2 Hz, ArH), 7.45 (t, 1H, J = 6.7 Hz, ArH), 7.2 (t, 1H, J = 6.5 Hz, ArH). ^13^CNMR (100 MHz, DMSO-d6) 150.33, 149.21, 140.00, 129.00, 126.11, 121.52, 73.00, 72.81, 72.33, 72.00, 54.69. HRMS (ESI) calcd for [C_9_H_9_N_2_O_3_]^+^: 193.13. Found: 193.11.

**5**-(**2**-**fluoro phenyl**)-**3H**-[**1**, **3**, **4**]-**oxadiazol**-**2**-**one** (**20**). IR (KBr) νmax: 3030.18, 2850.00, 1757.20, 1480.88, 1150.19, 1083.00, 1000.11, 800.10 cm^−1^. ^1^HNMR (400 MHz, DMSO-d6) δ 10.12 (brs, 1H, CONH),7.78–8.0 (m, 4H, ArH). ^13^CNMR (100 MHz, DMSO-d6) 163.11, 155.10, 154.67, 125.31,124.88, 123.78, 122.65, 122.10. HRMS (ESI) calcd for [C_8_H_6_FN_2_O_2_]^+^: 181.03. Found: 181.01.

**5**-[**4**-**methoxy phenyl**]-**3H**-[**1**, **3**, **4**]-**oxadiazol**-**2**-**one** (**21**). IR (KBr) νmax: 3021.03, 2894.10, 1760.10, 1479.45, 1160.30, 1075.01, 989.01,678.66 cm^−1^.^1^HNMR (400 MHz, DMSO-d6) δ 10.22 (brs, 1H,CONH), 7.65–7.80 (m, 4H, ArH), 7.40–7.60 (m, 2H, ArH). ^13^CNMR (100 MHz, DMSO-d6) 151.11, 149.79, 141.01, 121.35, 121.00, 120.35, 119.35, 114.60, 40.10. HRMS (ESI) calcd for [C_9_H_9_N_2_O_3_]^+^: 193.12. Found: 193.10.

**5**-(**4**-**mercapto phenyl**)-**3H**-[**1**, **3**, **4**]-**oxadiazol**-**2**-**one** (**22**). IR (KBr) νmax: 3010.01, 2489.12, 1758.10, 1485.18, 1165.13, 1087.01, 989.31 cm^−1^. ^1^HNMR (400 MHz, DMSO-d6) δ 10.00 (brs, 1H, CONH), 7.60–7.80 (m, 4H, ArH), 3.40 (s, 1H, ArSH). ^13^CNMR (100 MHz, DMSO-d6) 153.21, 152.98, 128.30, 124.14, 123.34, 122.56, 122.00, 113.13. HRMS (ESI) calcd for [C_8_H_7_N_2_O_2_S]^+^: 195.05. Found: 195.03.

**5**-(**4**-**nitro phenyl**)-**3H**-[**1**, **3**, **4**]-**oxadiazol**-**2**-**one** (**23**). IR (KBr) νmax: 3020.11,1749.26,1630 1486.38, 1157.12, 1090.02, 999.13 cm^−1^. ^1^HNMR (400 MHz, DMSO-d6) δ 10.010 (brs, 1H, CONH), 7.25–7.45 (m, 4H, ArH). ^13^CNMR (100 MHz, DMSO-d6) 153.26, 139.39, 129.10, 119.23, 118.98, 113.78, 113.00. HRMS (ESI) calcd for [C_8_H_6_N_3_O_4_]^+^: 208.07. Found: 208.00.

**5**-(**4**-**hydroxy phenyl**)-**3H**-[**1**, **3**, **4**]-**oxadiazol**-**2**-**one** (**24**). IR (KBr) νmax: 3550.13, 3010.23, 1758.20, 1481.98, 1170.18, 1210.00, 1079.00, 1000.21 cm^−1^. ^1^HNMR (400 MHz, DMSO-d6) δ 10.04 (brs, 1H, CONH), 8.8 (s, 1H, ArOH), 7.25–7.49 (m, 4H, ArH). ^13^CNMR (100 MHz, DMSO-d6) 154.78, 154.17, 142.33, 128.89, 128.00, 118.47, 117.33, 116.79. HRMS (ESI) calcd for [C_8_H_7_N_2_O_3_]^+^: 179.11. Found: 179.02.

**5**-(**3**,**5 dimethoxy phenyl**)-**3H**-[**1**, **3**, **4**]-**oxadiazol**-**2**-**one** (**25**). IR (KBr) νmax: 3020, 2856, 1761.11, 1485.77, 1160.20, 1084.00, 1001.11 cm^−1^. ^1^HNMR (400 MHz, DMSO-d6) δ 10.00 (brs, 1H,CONH), 8.1(s, 1H, J = 7.8 Hz, ArH), 7.30–7.40 (m, 2H, ArH), 3.8 (s, 6H, 2xArOCH_3_). ^13^CNMR (100 MHz, DMSO-d6) 155.03, 154.98, 144.00, 143.79, 134.37, 118.00, 117.66, 117.00, 53.00, 52.97. HRMS (ESI) calcd for [C_10_H_11_N_2_O_4_]^+^: 223.14. Found: 223.06.

**5**-(**3**-**trifluoro phenyl**)-**3H**-[**1**, **3**, **4**]-**oxadiazol**-**2**-**one** (**26**). IR (KBr) νmax: 3019, 1753.33, 1488.89, 1159.20, 1079.00, 1060.15 cm^−1^. ^1^HNMR (400 MHz, DMSO-d6) δ 9.89 (brs, 1H, CONH), 8.4 (m, 1H, ArH),8.0 (m, 1H, ArH),7.89 (s, 1H, ArH), 7.45 (m, 1H, ArH). ^13^CNMR (100 MHz, DMSO-d6) 155.32, 154.66,129.10,121.36, 121.00, 120.55, 120.00, 115.11. HRMS (ESI) calcd for [C_9_H_6_F_3_N_2_O_2_]^+^: 231.00. Found: 231.10.

**5**-(**4**-**methyl phenyl**)-**3H**-[**1**, **3**, **4**]-**oxadiazol**-**2**-**one** (**27**). IR (KBr) νmax: 3018, 2859, 1760.20, 1481.11, 1153.16, 1078.23, 1010.15 cm^−1^. ^1^HNMR (400 MHz, DMSO-d6) δ 10.10 (brs, 1H,CONH), 8.20 (d, J = 8.8 Hz, 2H, ArH), 7.88 (d, J = 8.00, 2H, ArH), 2.30 (s, 3H,ArCH_3_). ^13^CNMR (100 MHz, DMSO-d6) 159.00, 158.30, 129.90, 122.88, 121.89, 120.98, 119.89, 112.79. HRMS (ESI) calcd for [C_9_H_9_N_2_O_2_]^+^: 177.23. Found: 177.34.

**5**-(**3**-**fluoro**, **4**-**methyl phenyl**)-**3H**-[**1**, **3**, **4**]-**oxadiazol**-**2**-**one** (**28**). IR (KBr) νmax: 3013, 2869, 1755.44, 1486.32, 1157.16, 1081.00, 1020.19 cm^−1^. ^1^HNMR (400 MHz, DMSO-d6) δ 10.40 (brs, 1H,CONH), 8.34 (s, 1H, ArH),7.80 (d, 2H, J = 7.6 Hz, ArH), 7.64 (d,1H, J = 7.4 Hz, ArH), 2.21 (s, 3H, ArCH_3_). ^13^CNMR (100 MHz, DMSO-d6) 164.69, 150.10, 149.88,123.22, 122.00, 121.03, 120.33, 114.23, 22.00. HRMS (ESI) calcd for [C_9_H_8_FN_2_O_2_]^+^: 195.31. Found: 195.10.

**5**-(**3**,**5**-**difluoro phenyl**)-**3H**-[**1**, **3**, **4**]-**oxadiazol**-**2**-**one** (**29**). IR (KBr) νmax: 3030, 1760.25, 1479.72, 1149.20, 1063.00, 1010.16 cm^−1^. ^1^HNMR (400 MHz, DMSO-d6) δ 10.42 (brs, 1H,CONH), 8.46 (s, 1H, ArH), 8.20 (s, 2H, ArH). ^13^CNMR (100 MHz, DMSO-d6) 163.10, 162.89, 151.10, 150.22, 126.10, 118.15, 118.00, 117.67. HRMS (ESI) calcd for [C_8_H_5_F_2_N_2_O_2_]^+^: 199.17. Found: 199.10.

## Electronic supplementary material

Supplementary information.
