# Supplementary material for: Sulfonated reduced graphene oxide catalyzed cyclization of hydrazides and carbon dioxide to 1,3,4-oxadiazoles under sonication
Source: Sci Rep. 2017 Jul 5;7:4675. doi: 10.1038/s41598-017-04143-4 (PMC5498654; doi:10.1038/s41598-017-04143-4)
Supplement: Supplementary file 1 — Supplementary information. [file 41598_2017_4143_MOESM1_ESM.pdf]

Supplementary information

Sulfonated reduced graphene oxide catalyzed cyclization of hydrazides and carbon dioxide to 1,3,4-oxadiazoles under sonication

Manuri Brahmayya, Shenghong A Dai, Shing-Yi Suen*

Department of Chemical Engineering, National Chung Hsing University, 250 KuoKuang Raod 250, Taichung, Taiwan, R.O.C

Correspondence author : *E-mail:* sysuen@dragon.nchu.edu.tw

Table S1. Cyclization of 4-methyl benzohydrazide under various conditions.

Temperature / Sonication	Time (h)	5 wt% catalyst loading		20 wt% catalyst loading		50 wt% catalyst loading	
		$PhCONHNH_2$	POxdO	$PhCONHNH_2$	POxdO	$PhCONHNH_2$	POxdO
25 °C / Conventional	3	100	0	93.4	6.6	91.6	8.4
	6	100	0	87.2	12.8	85.5	14.5
	24	99.8	0	78.8	20.2	79.2	20.8
	48	99.0	1.0	76.6	23.4	75.0	25.0
	72	99.0	1.0	71.3	28.7	69.9	30.1
	96	98.1	1.8	63.7	36.3	61.7	38.3
	**Time (min)**	**5 wt% catalyst loading**		**20 wt% catalyst loading**		**50 wt% catalyst loading**	
		**$PhCONHNH_2$**	**POxdO**	**$PhCONHNH_2$**	**POxdO**	**$PhCONHNH_2$**	**POxdO**
25 °C / Sonication	10	99.0	1.0	93.0	7.0	92.3	7.7
	30	94.5	5.5	83.6	16.4	81.2	18.8
	50	94.0	6.0	69.7	30.3	65.0	35.0
	60	93.0	7.0	66.0	34.0	63.6	36.4
	90	92.8	7.2	63.2	36.8	62.1	37.9
	120	92.8	7.1	61.0	39.0	60.0	40
40 °C / Sonication	10	98.0	2.0	89.9	10.1	88.2	11.8
	30	97.4	2.6	81.8	18.2	76.8	23.2
	50	92.6	7.4	67.5	32.5	63.0	37.0
	60	91.8	8.2	63.9	36.1	62.2	37.8
	90	91.0	8.2	63.0	37.0	61.0	39.0
	120	92.0	8.0	60.6	39.4	60.4	39.6
50 °C / Sonication	10	97.0	3.0	89.1	10.9	88.0	12.0
	30	93.0	7.0	74.5	25.5	73.0	27.0
	50	90.1	9.9	62.0	38.0	70.2	29.8
	60	90.0	9.0	59.7	40.3	68.1	41.9
	90	90.3	9.7	59.0	41.0	57.7	42.3
	120	90.7	9.3	58.9	41.1	57.2	42.8
75 °C / Sonication	10	97.1	1.9	91.2	9.8	89.5	10.5
	30	92.8	1.5	83.2	16.8	79.8	20.2
	50	93.6	6.4	70.0	30.0	70.0	30.0
	60	90.3	9.7	72.4	27.6	69.3	30.7
	90	90.6	9.4	73.6	26.4	70.0	30.0
	120	90.8	8.2	74.0	26.0	71.0	29.0
100 °C / Sonication	10	98.0	4.0	92.0	8.0	91.0	9.0
	30	96.2	4.0	87.0	13.0	85.0	15.0
	50	95.1	4.9	79.1	20.9	77.7	22.3
	60	95.0	5.0	79.6	20.4	78.1	21.9
	90	95.0	9.7	80.9	19.1	80.0	20.0
	120	96.0	9.3	81.3	18.7	80.2	19.8

[a] All reactions employed in sealed, Teflon-lined 25 mL round bottomed flasks. Aliquots were picked out at time intervals listed in the table s1. The products were extracted in DMSO-d_6 (1 mL) and separated from the nano catalyst by the syringe filtration (0.2 μm PTFE). Conversions were checked by integration of the hydrazide peak (CONHNH) of 4-methyl benzohydrazide or the ortho cyclic 1,3,4-oxadiazole of CONH.

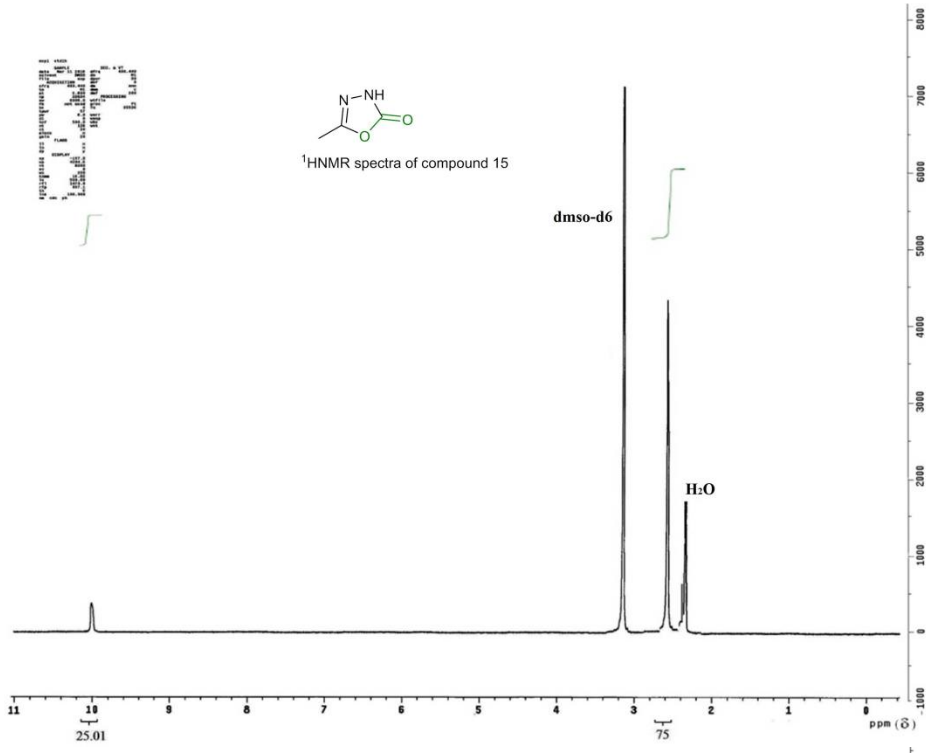

N–NH
O
O
^{1}HNMR spectra of compound 15
dmso-d6
H₂O
11 10 9 8 7 6 5 4 3 2 1 0
ppm (δ)
25.01
75
8000 7000 6000 5000 4000 3000 2000 1000 0 -1000

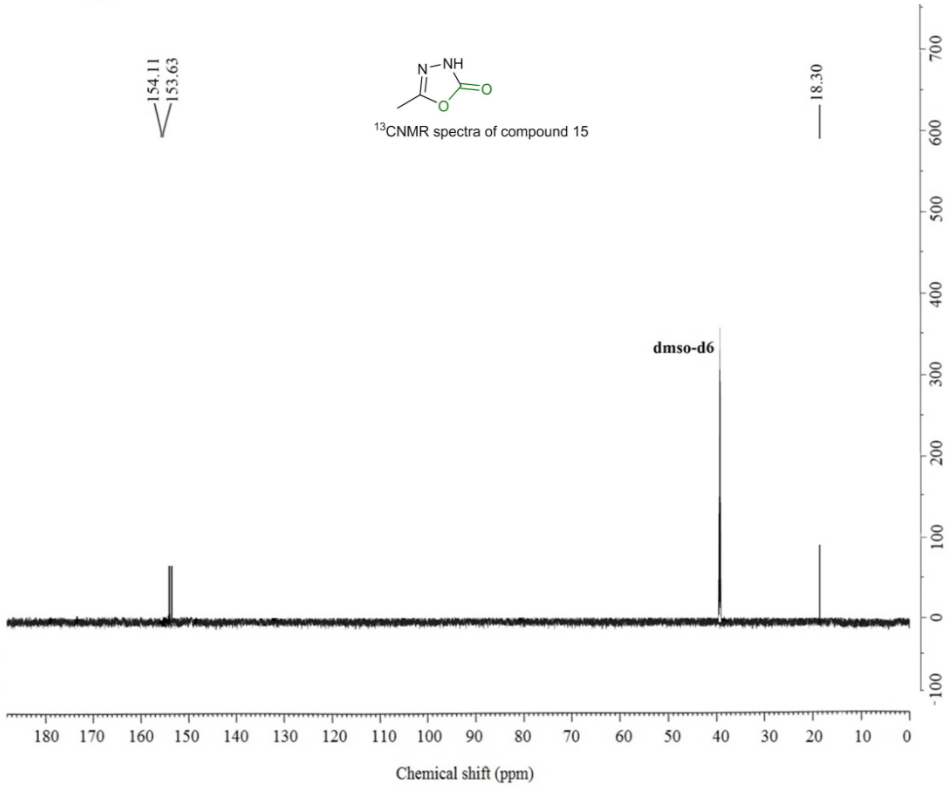

154.11
153.63
18.30
N–NH
O
O
^{13}CNMR spectra of compound 15
dmso-d6
700 600 500 400 300 200 100 0 -100
180 170 160 150 140 130 120 110 100 90 80 70 60 50 40 30 20 10 0
Chemical shift (ppm)

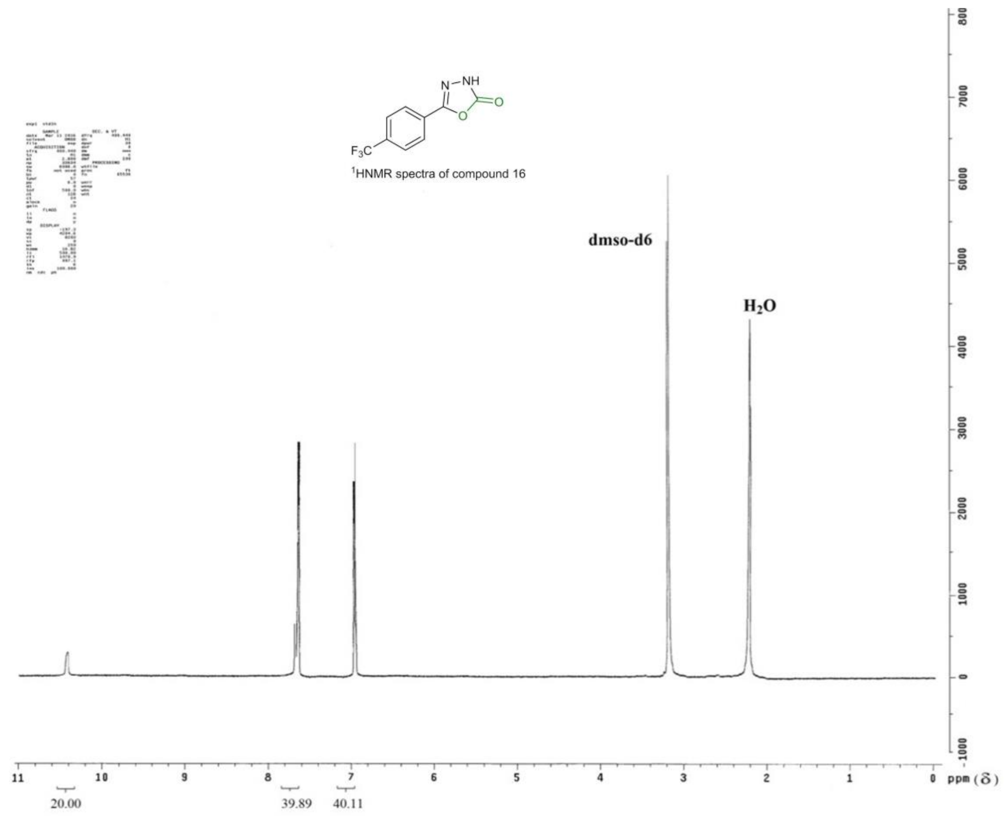

N
NH
O
O
F3C
1HNMR spectra of compound 16
dmso-d6
H2O
11 10 9 8 7 6 5 4 3 2 1 0 ppm (δ)
20.00
39.89
40.11

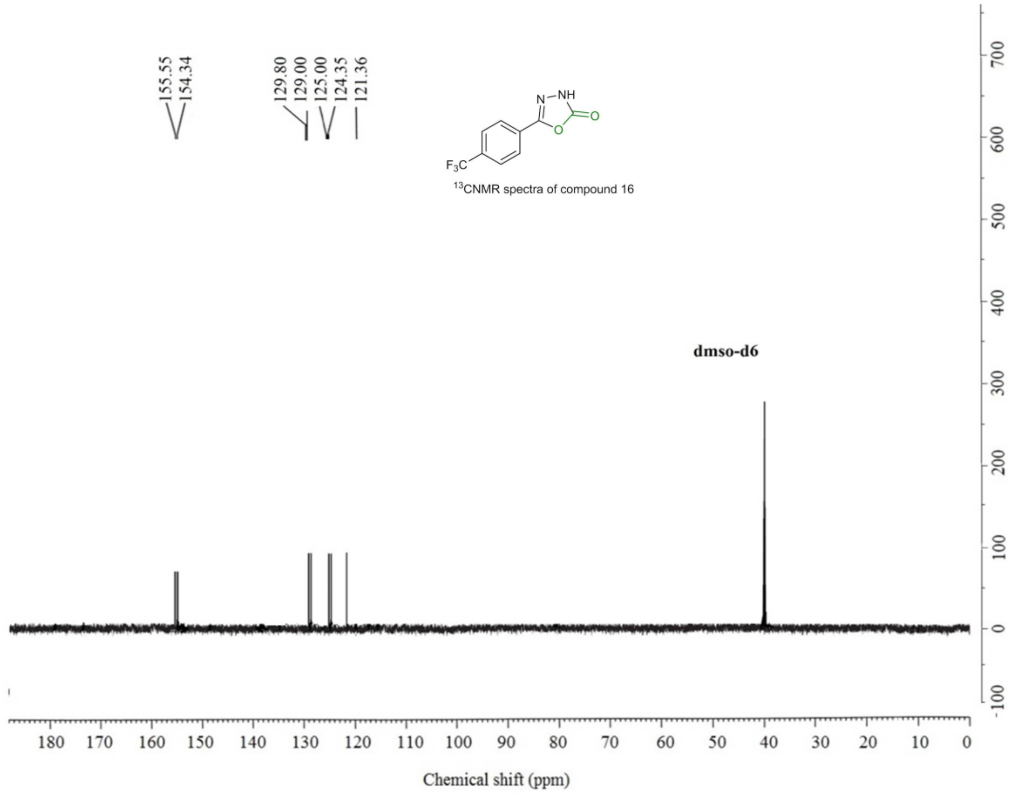

155.55
154.34
129.80
129.00
125.00
124.35
121.36
N
NH
O
O
F3C
13CNMR spectra of compound 16
dmso-d6
180 170 160 150 140 130 120 110 100 90 80 70 60 50 40 30 20 10 0
Chemical shift (ppm)

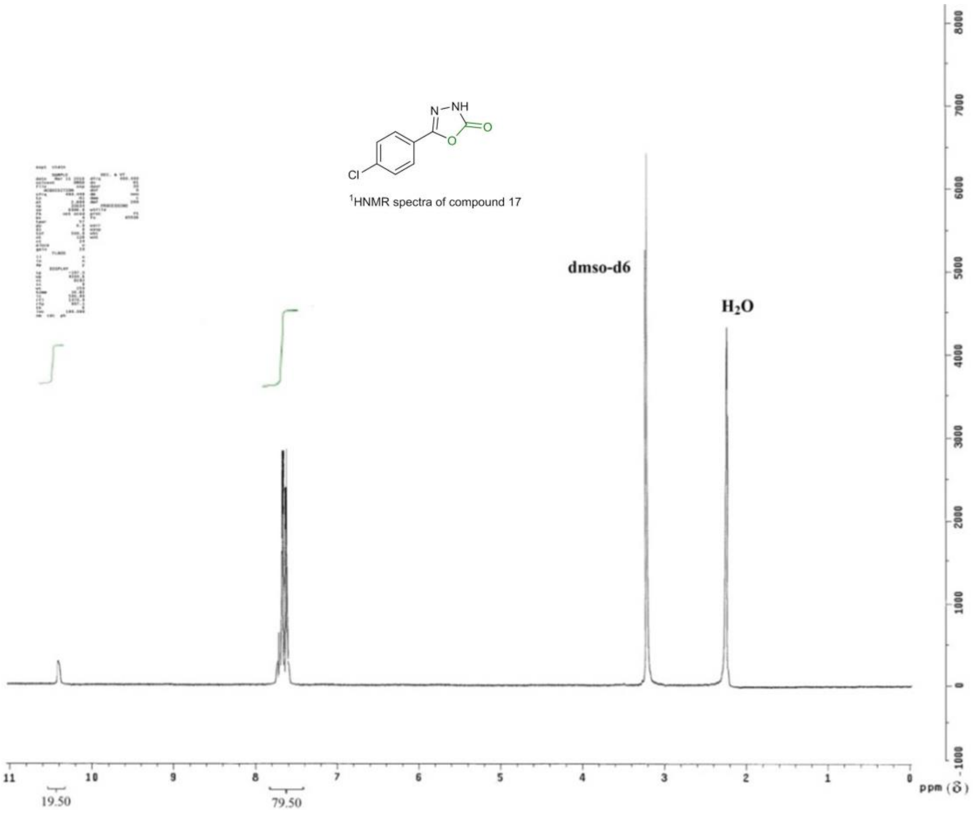

^{1}HNMR spectra of compound 17

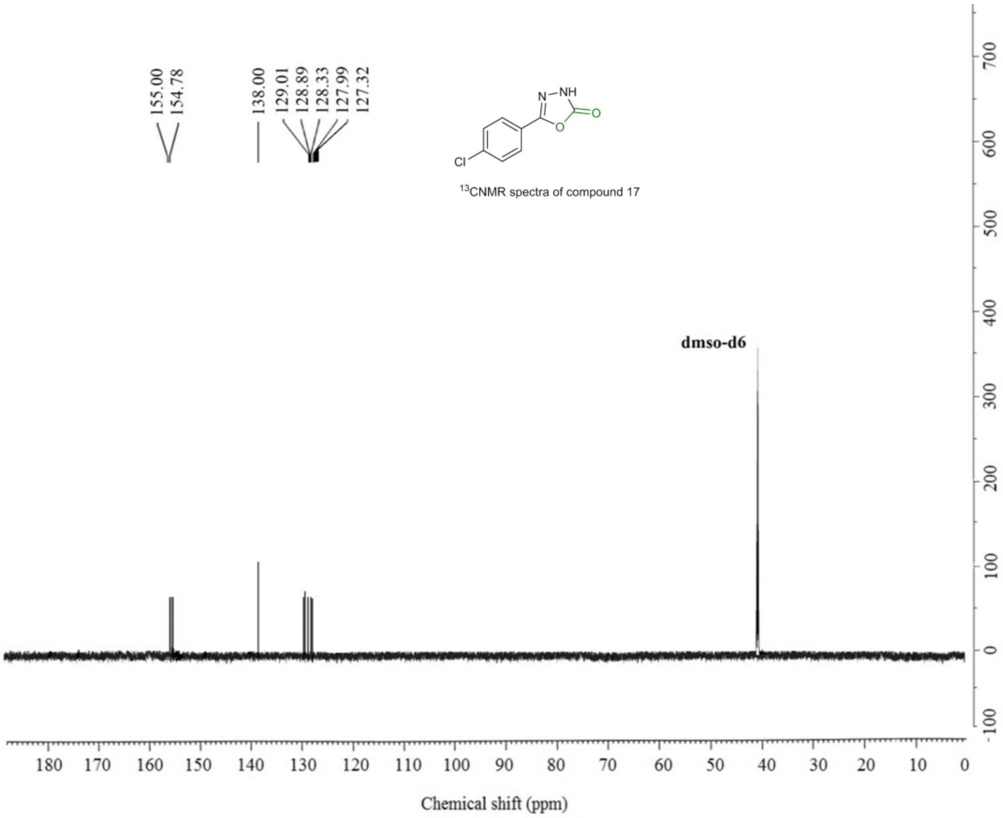

^{13}CNMR spectra of compound 17

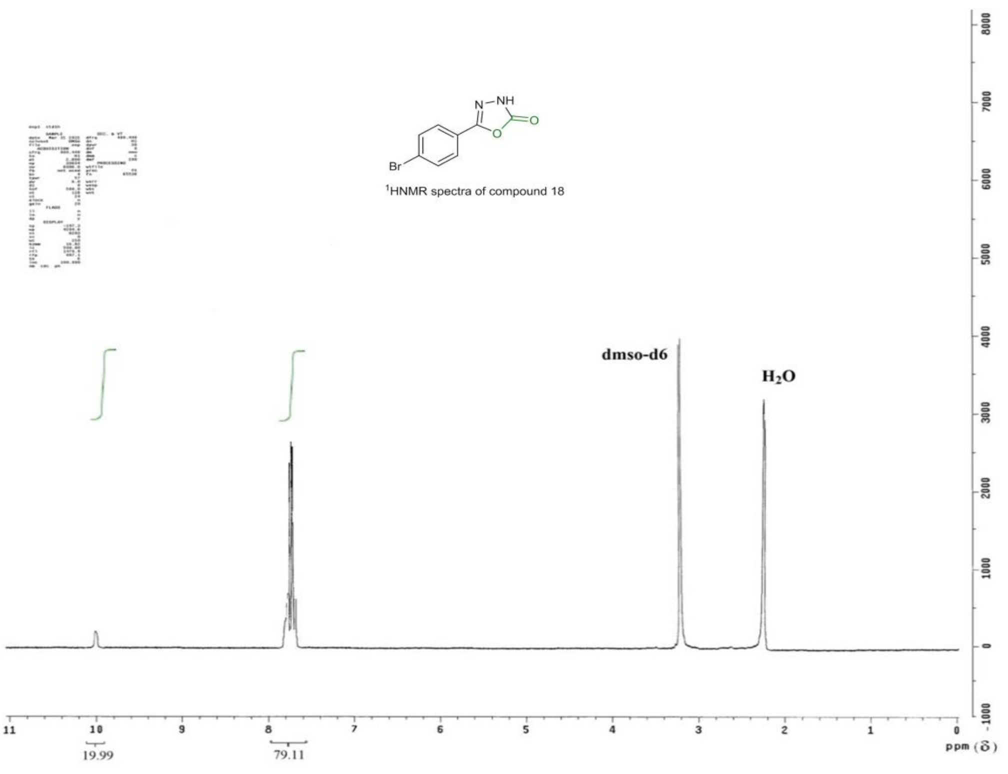

N
NH
O
O
Br
^{1}HNMR spectra of compound 18
dmso-d6
H_2O
19.99
79.11
ppm (δ)

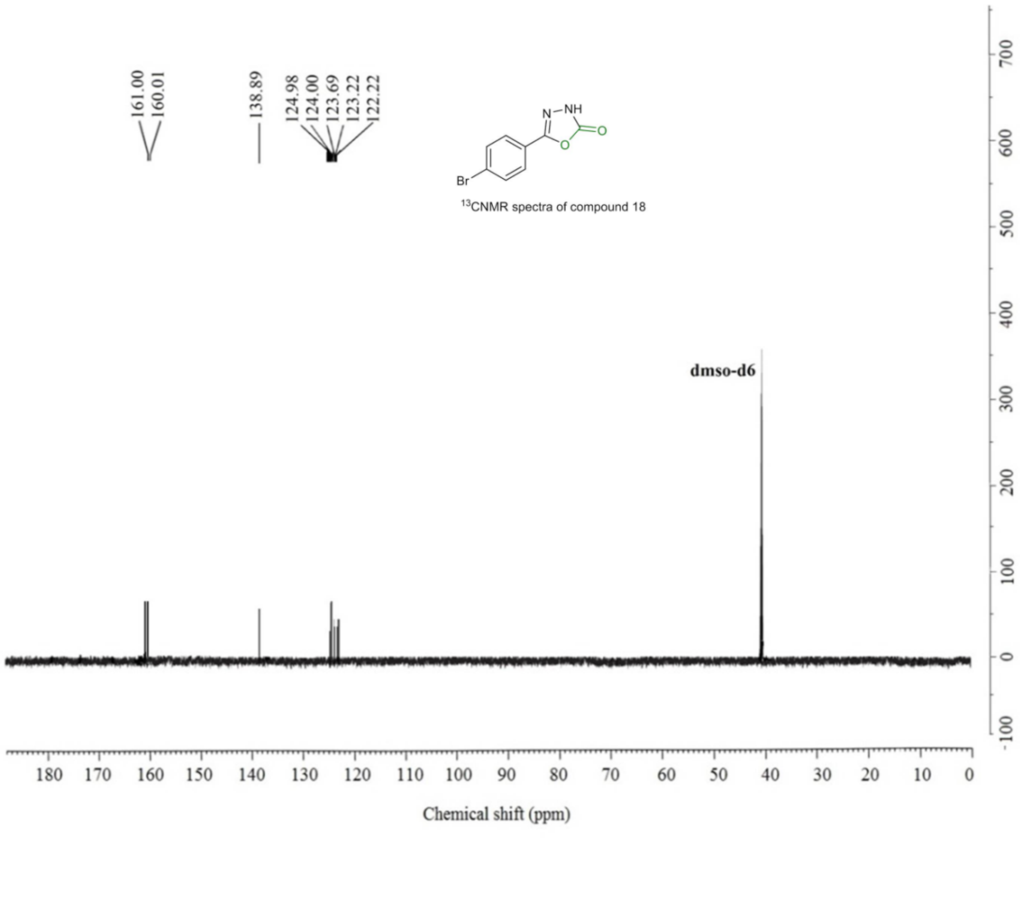

161.00
160.01
138.89
124.98
124.00
123.69
123.22
122.22
N
NH
O
O
Br
^{13}CNMR spectra of compound 18
dmso-d6
Chemical shift (ppm)

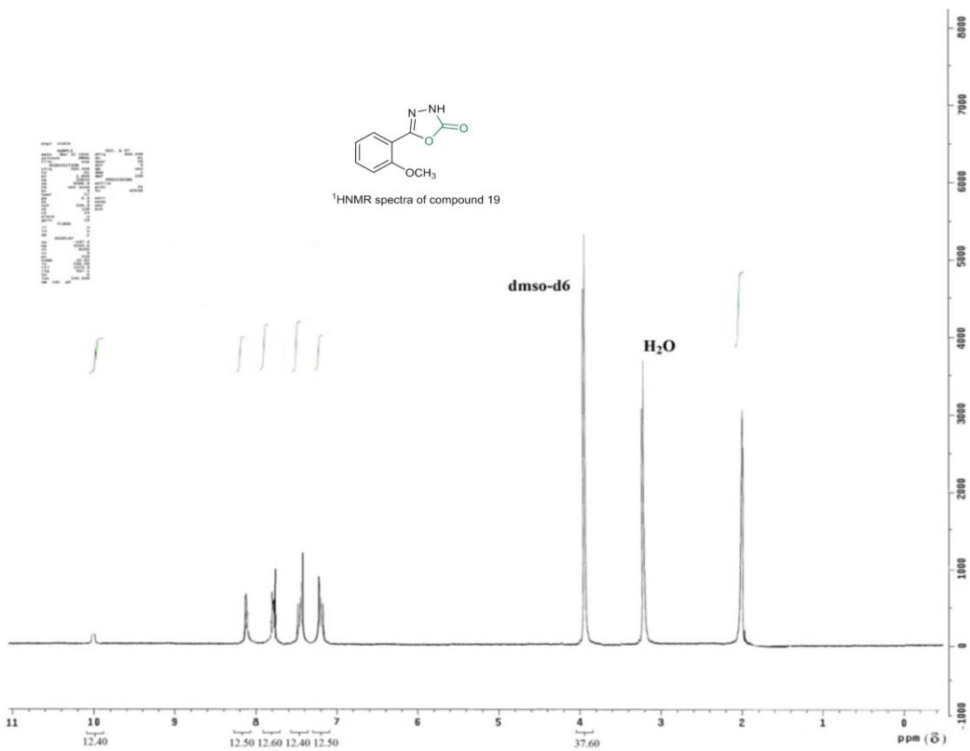

^{1}HNMR spectra of compound 19

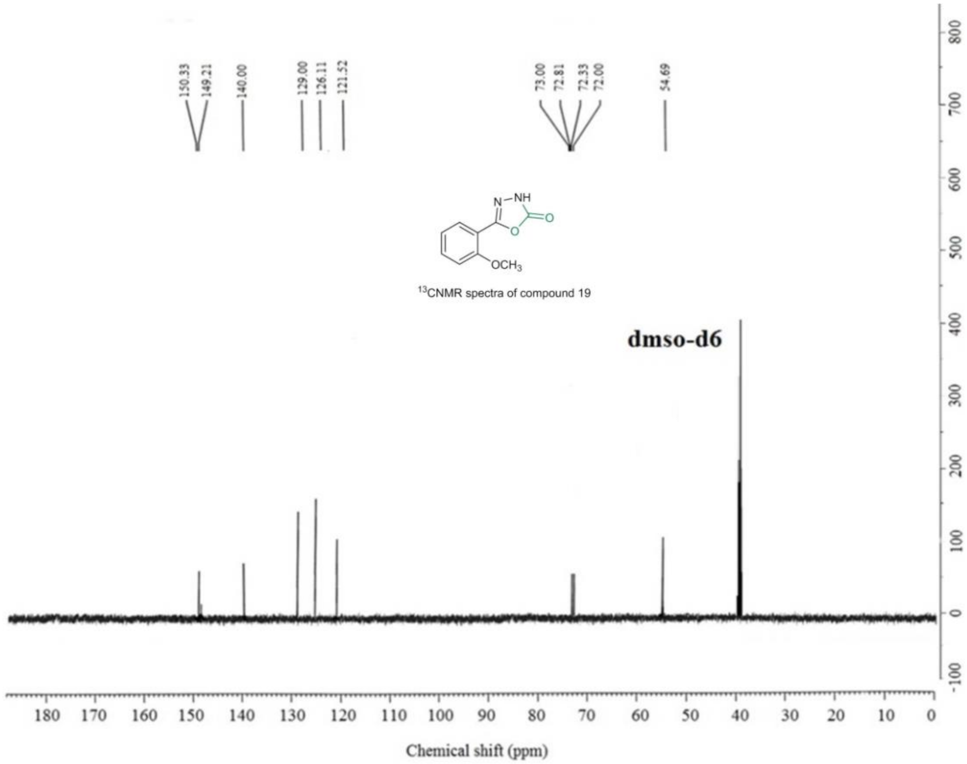

^{13}CNMR spectra of compound 19

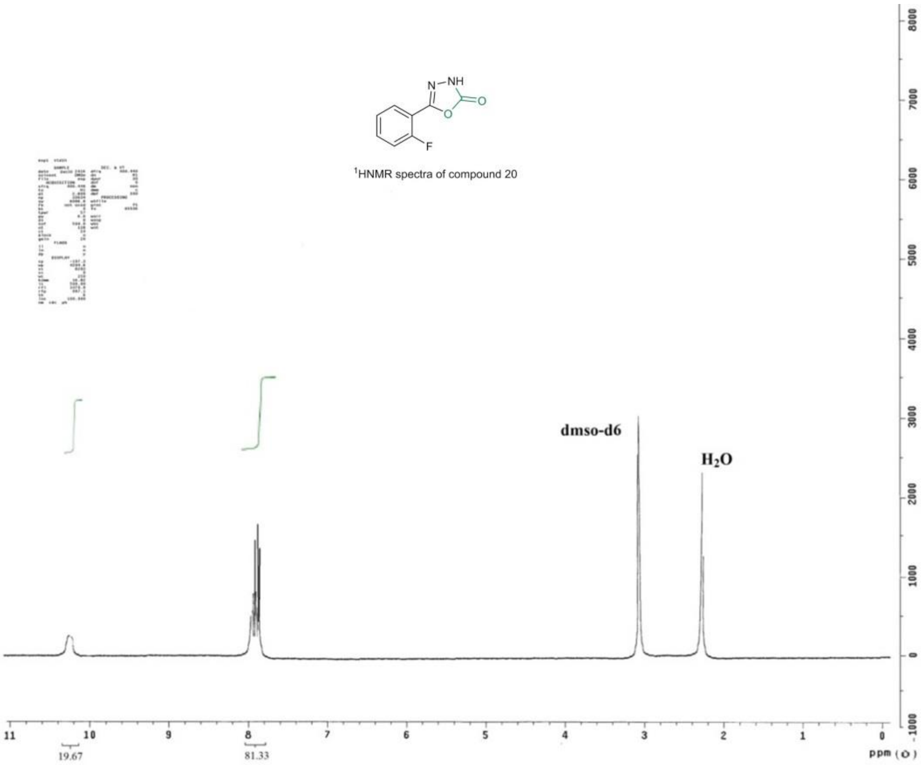

^{1}HNMR spectra of compound 20

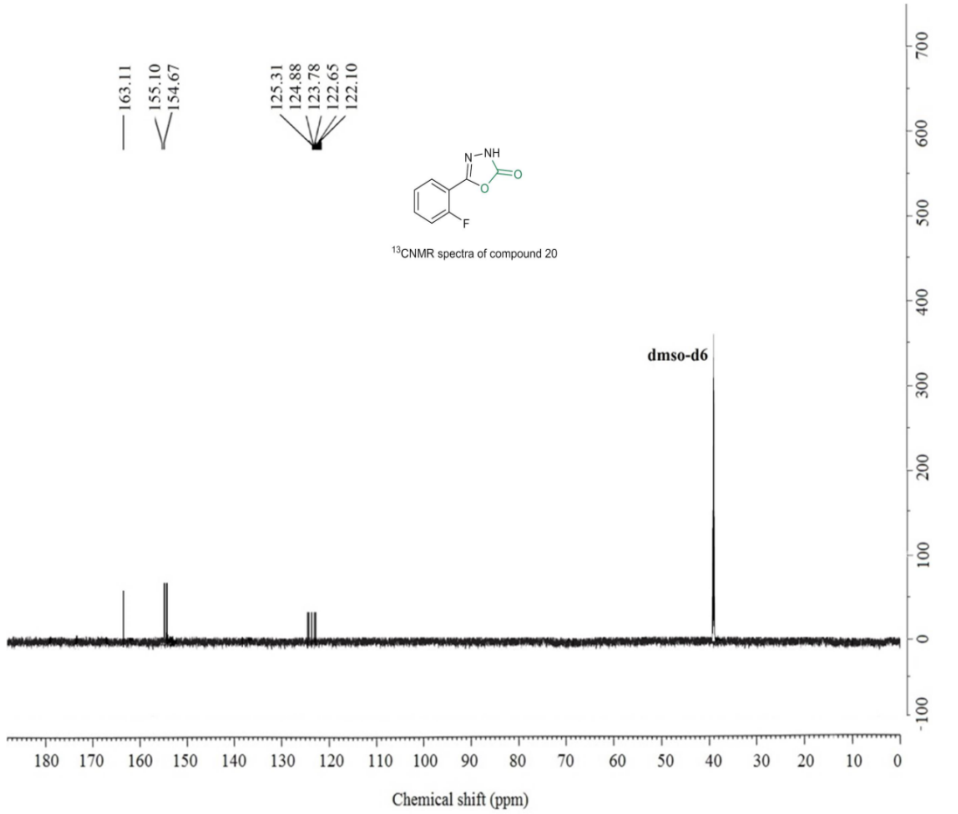

^{13}CNMR spectra of compound 20

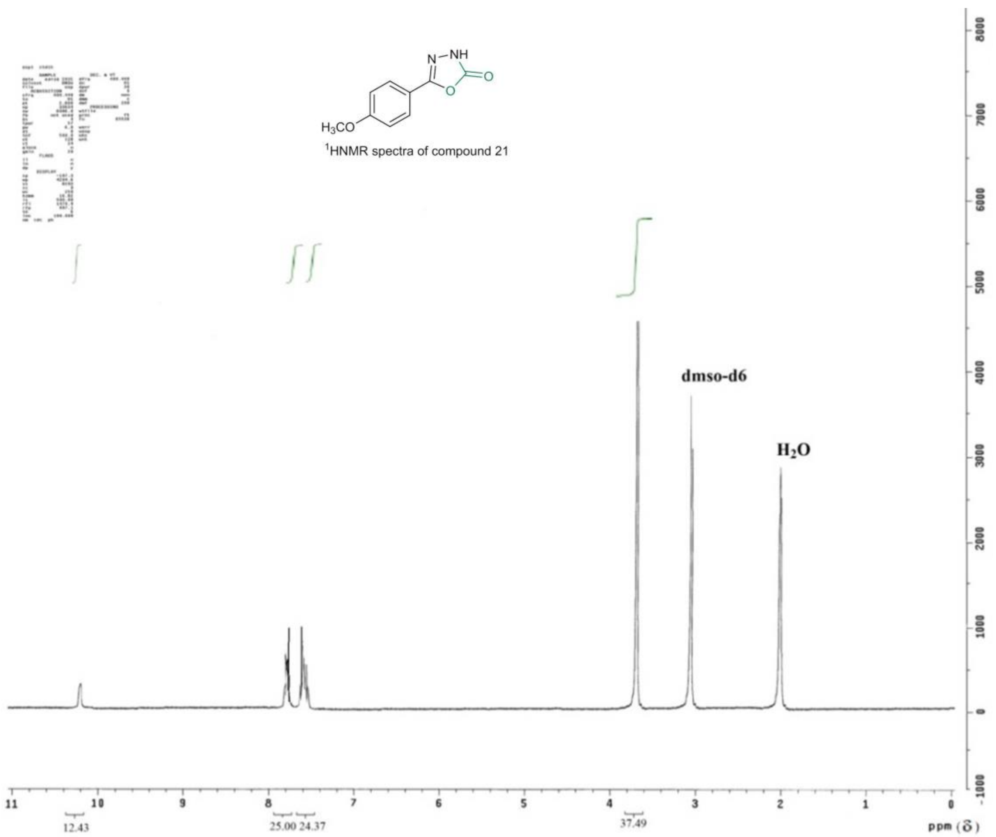

^{1}HNMR spectra of compound 21

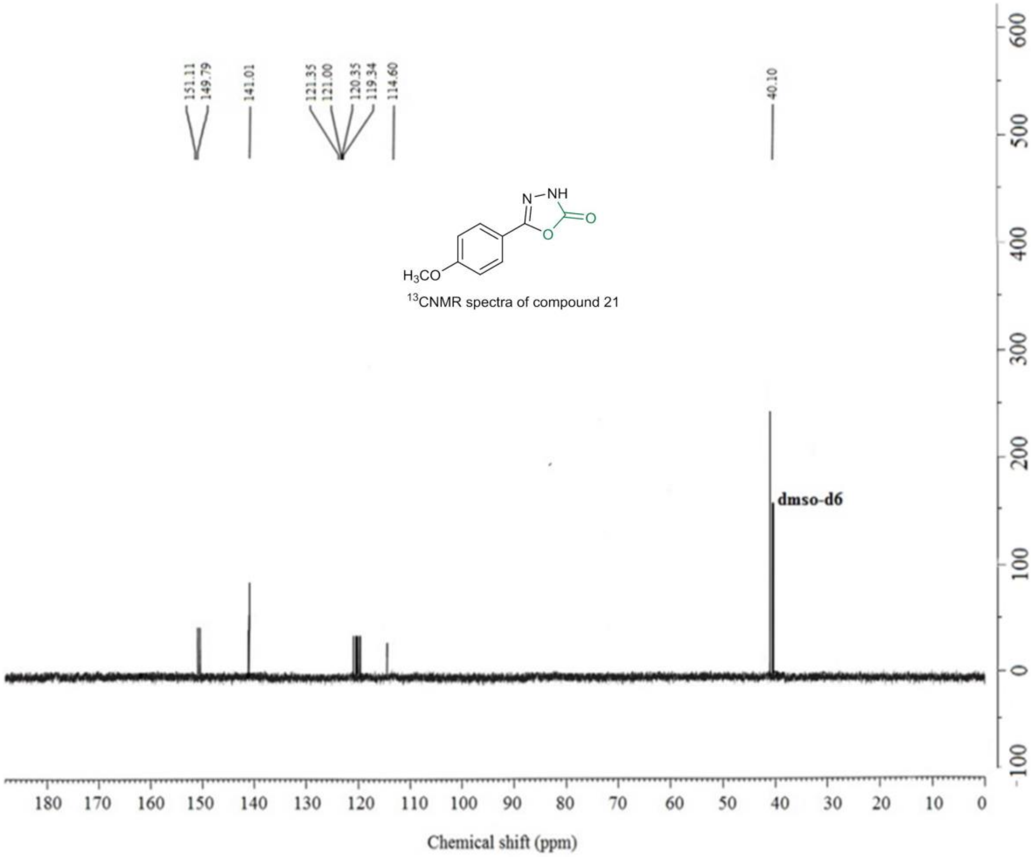

^{13}CNMR spectra of compound 21

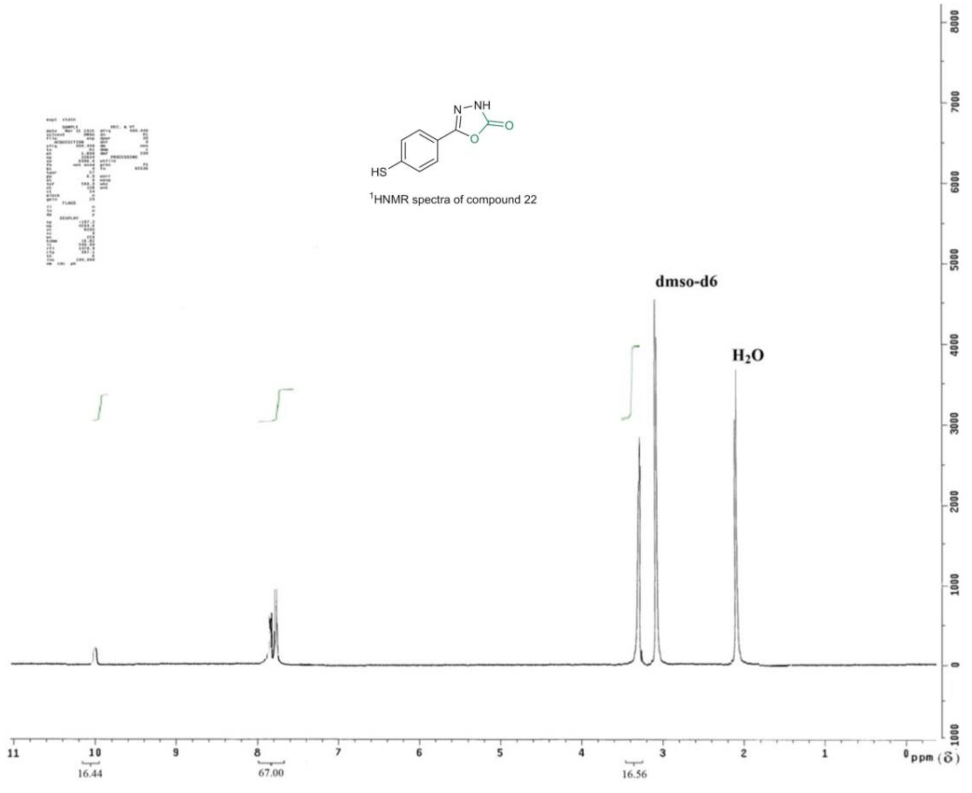
N–NH
HS
O
O
^{1}HNMR spectra of compound 22
dmso-d6
H2O
16.44
67.00
16.56
ppm (δ)

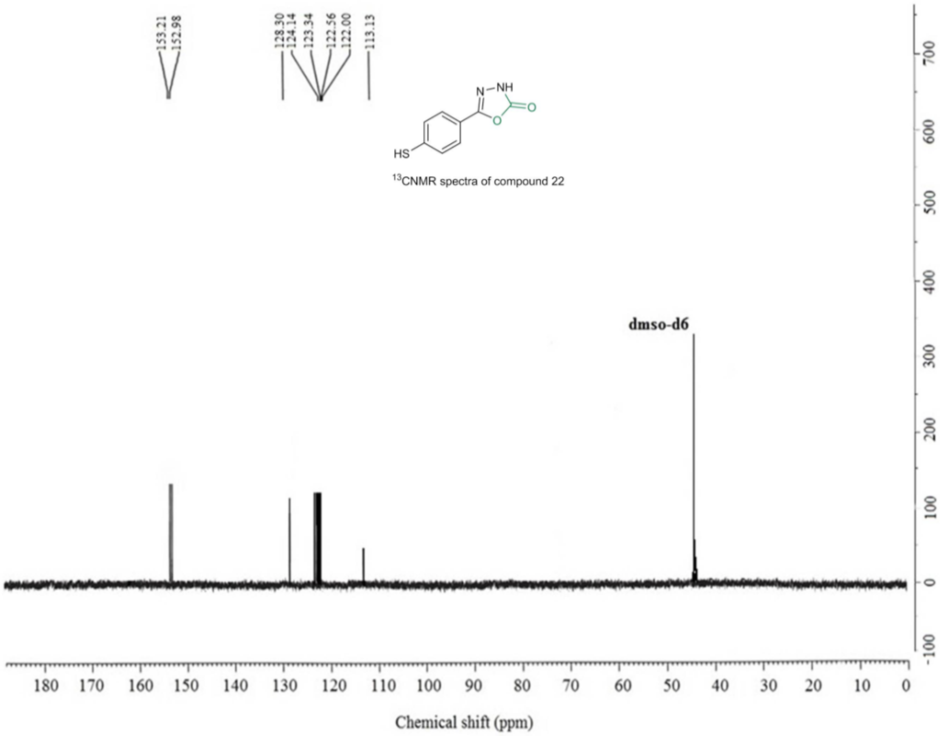
153.21
152.98
128.30
124.14
123.34
122.56
122.00
113.13
N–NH
HS
O
O
^{13}CNMR spectra of compound 22
dmso-d6
Chemical shift (ppm)

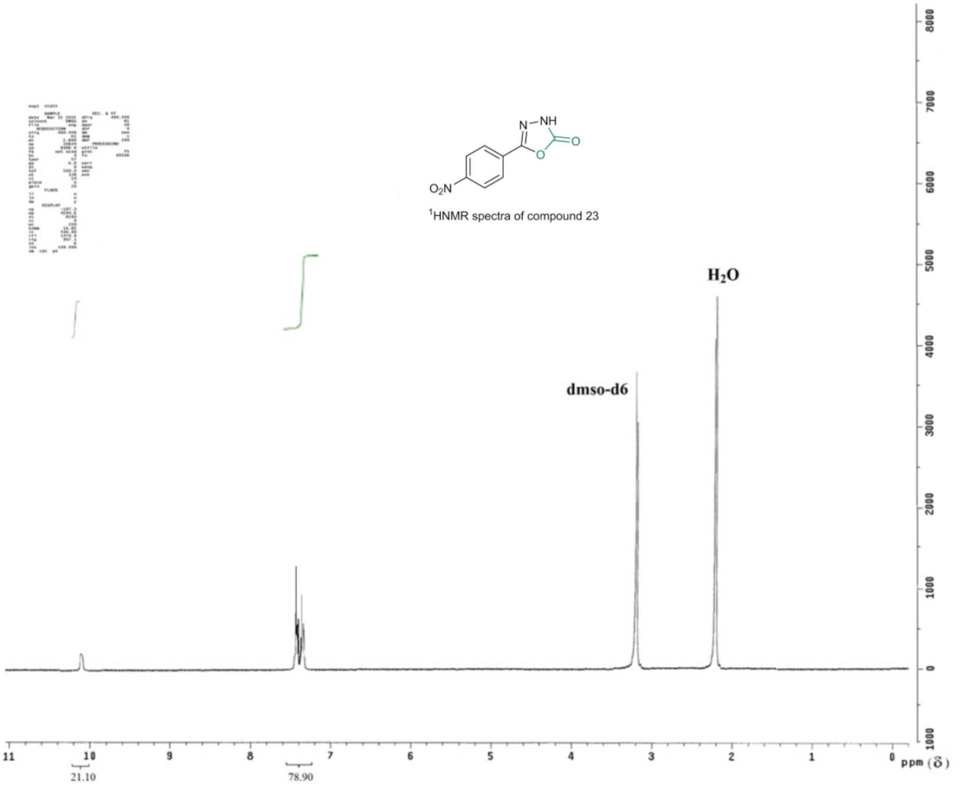

^{1}HNMR spectra of compound 23

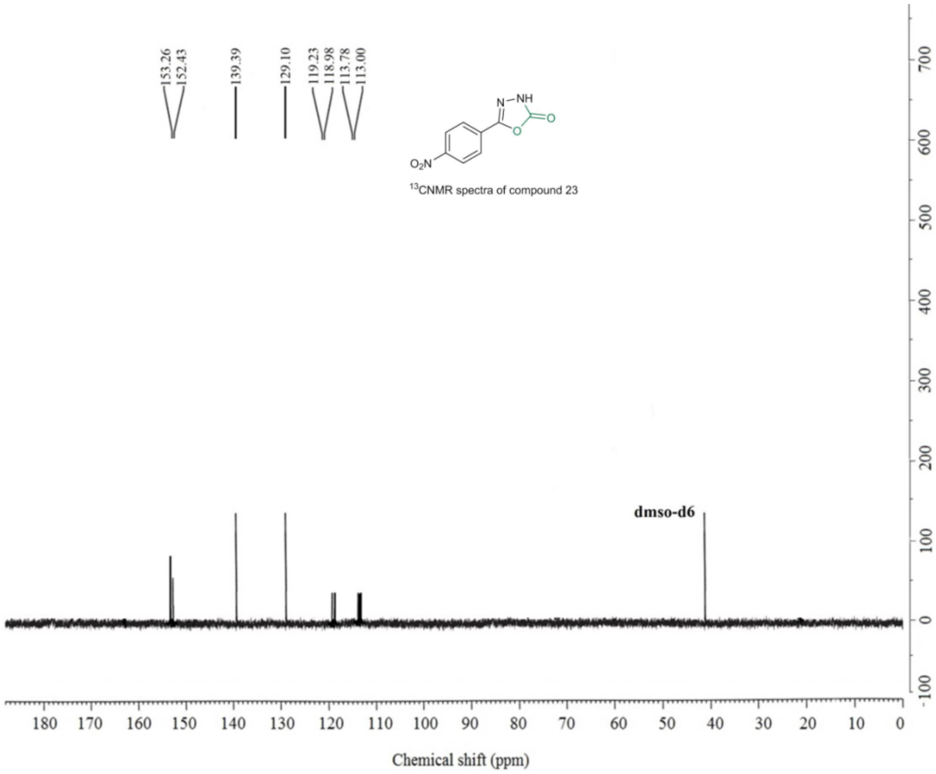

^{13}CNMR spectra of compound 23

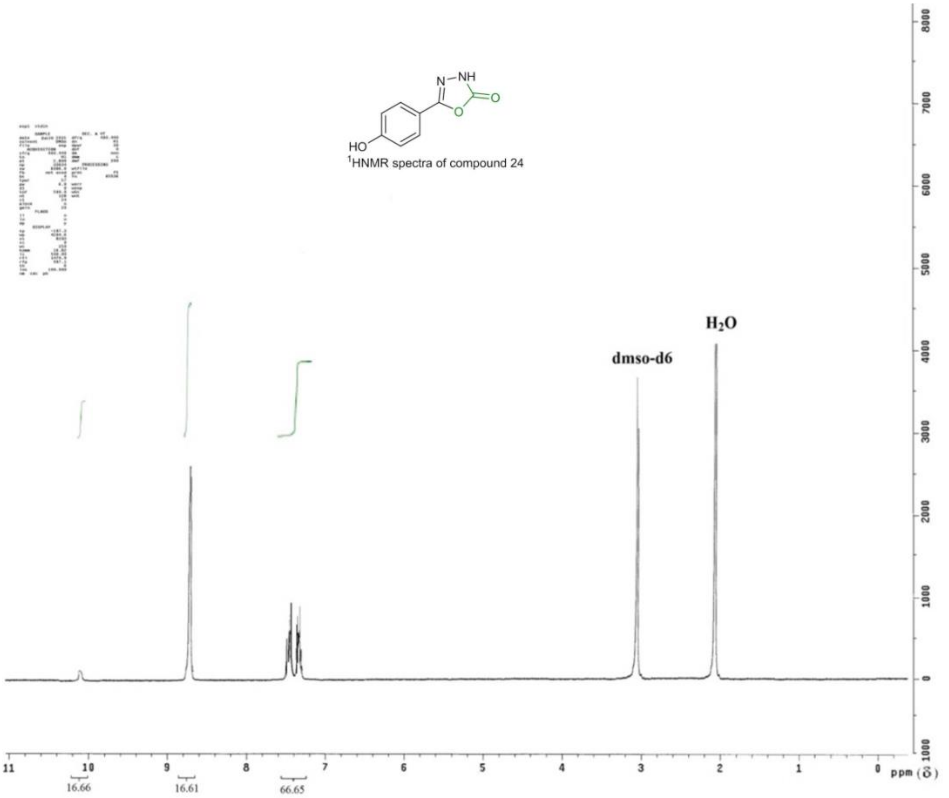
N–NH
O
HO
¹HNMR spectra of compound 24
dmso-d6
H_2O
16.66
16.61
66.65
ppm (δ)

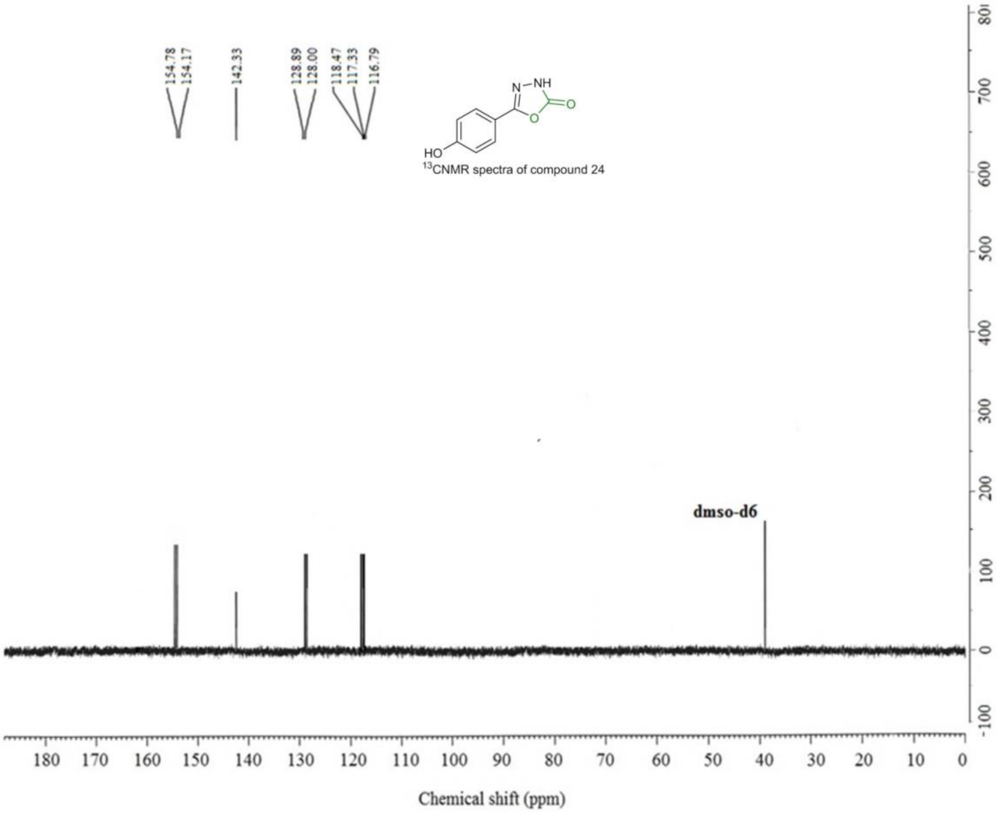
154.78
154.17
142.33
128.89
128.00
118.47
117.33
116.79
N–NH
O
HO
¹³CNMR spectra of compound 24
dmso-d6
Chemical shift (ppm)

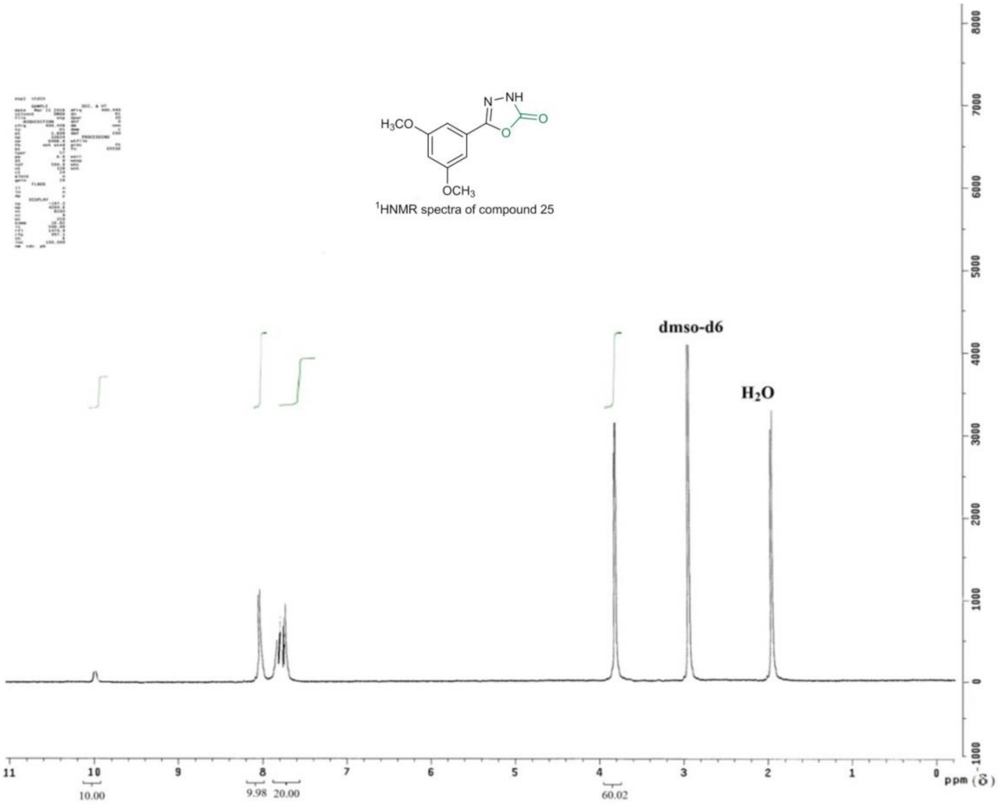
H3CO
N–NH
O
OCH3
1HNMR spectra of compound 25
dmso-d6
H2O
10.00
9.98
20.00
60.02
ppm (δ)

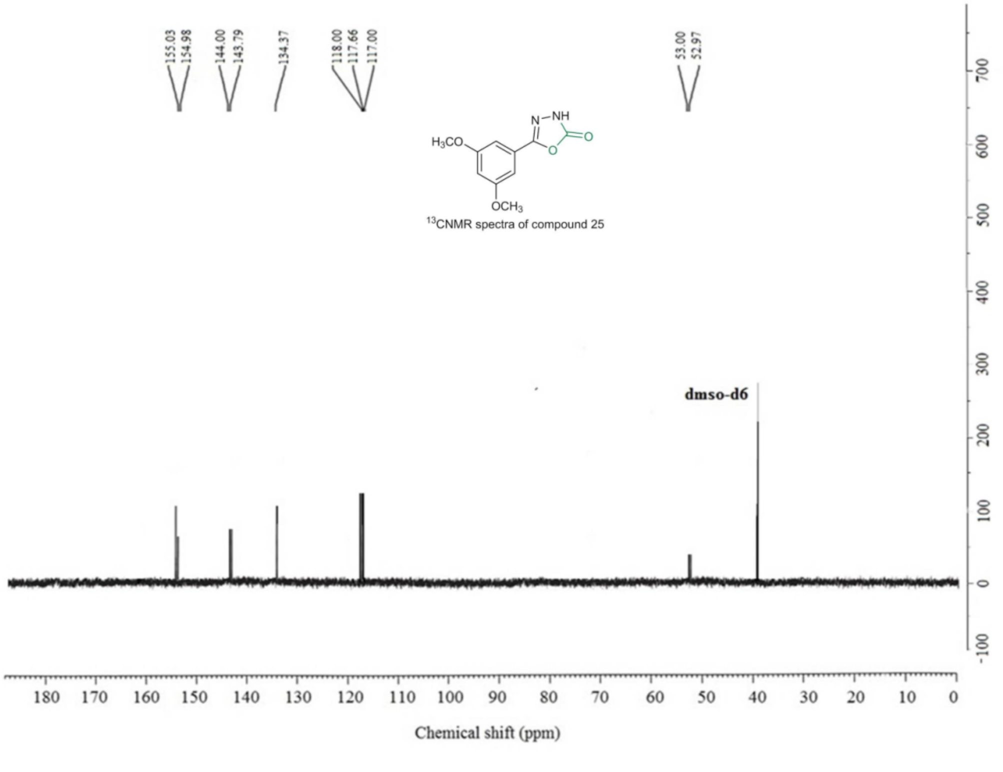
155.03
154.98
144.00
143.79
134.37
118.00
117.66
117.00
53.00
52.97
H3CO
N–NH
O
OCH3
13CNMR spectra of compound 25
dmso-d6
Chemical shift (ppm)

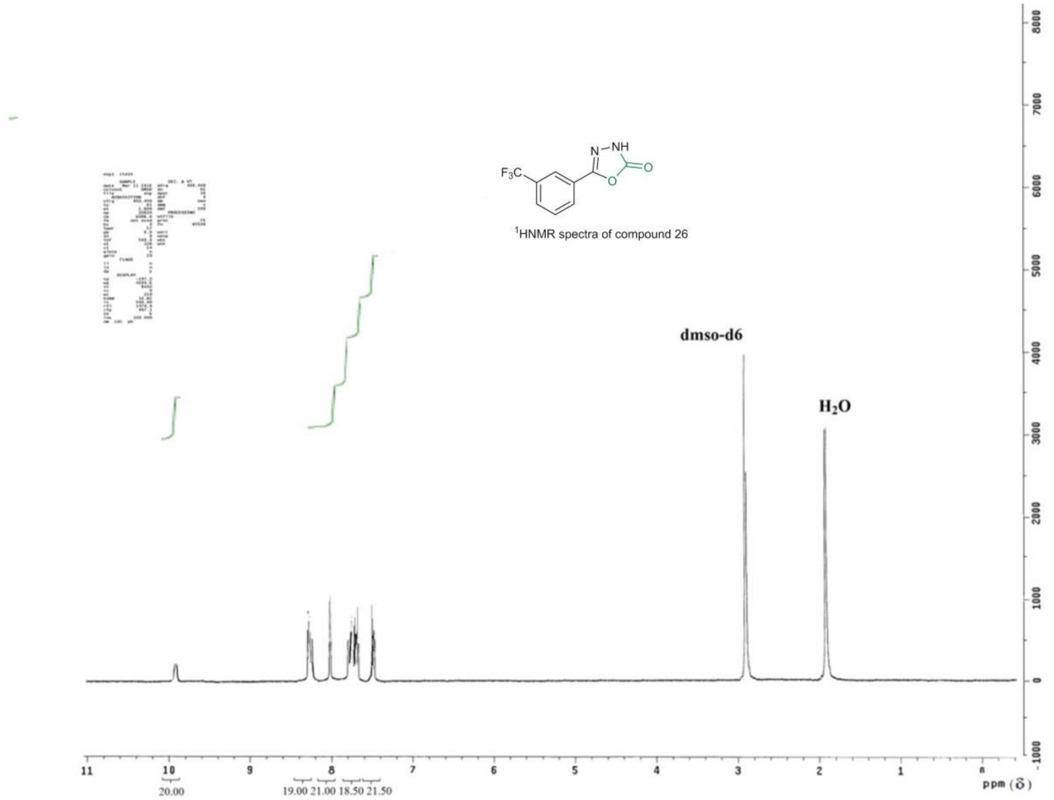

^{1}HNMR spectra of compound 26

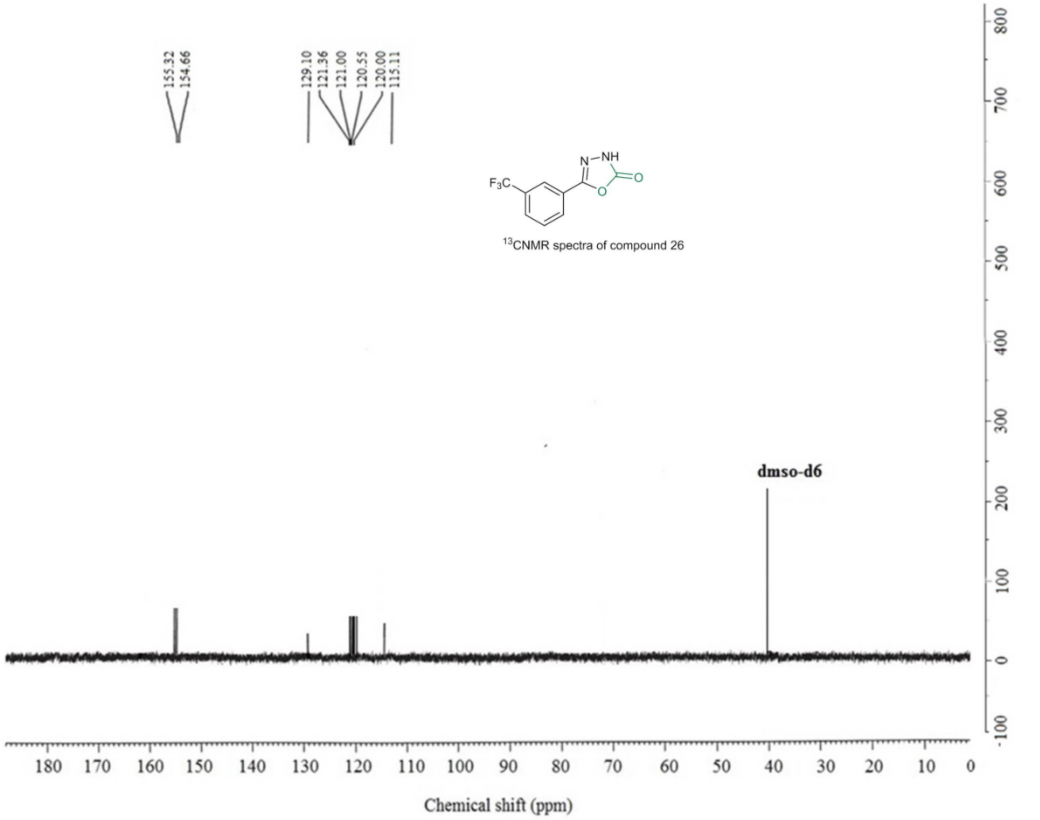

^{13}CNMR spectra of compound 26

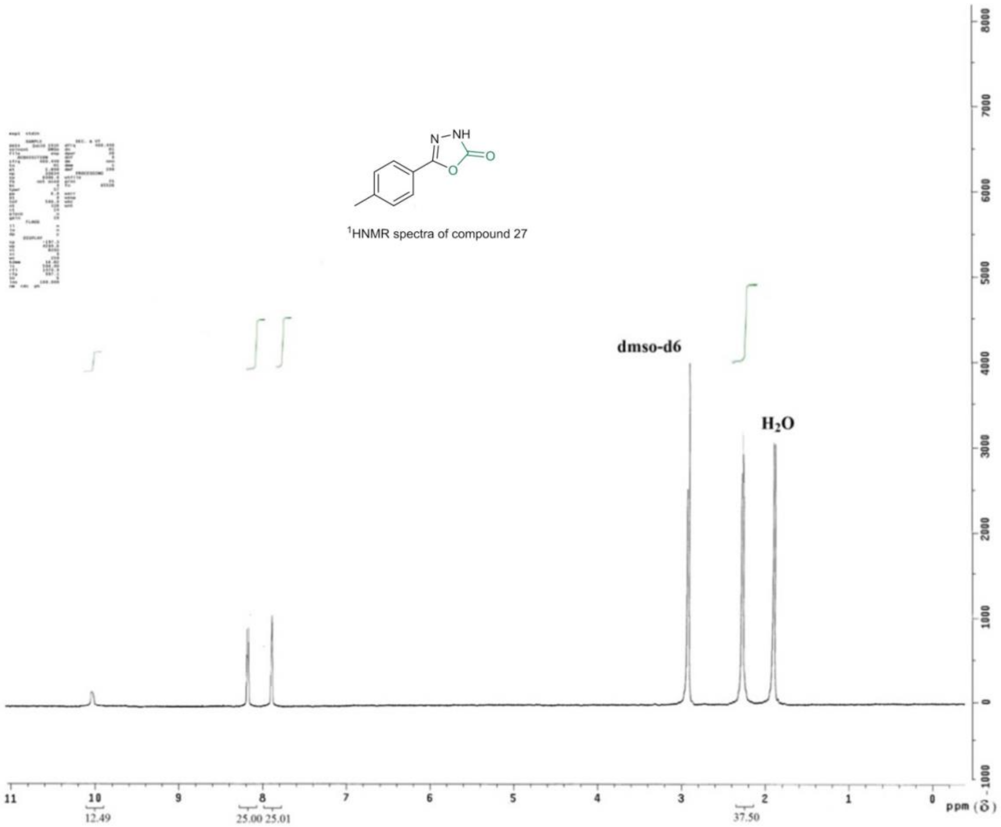

^{1}HNMR spectra of compound 27

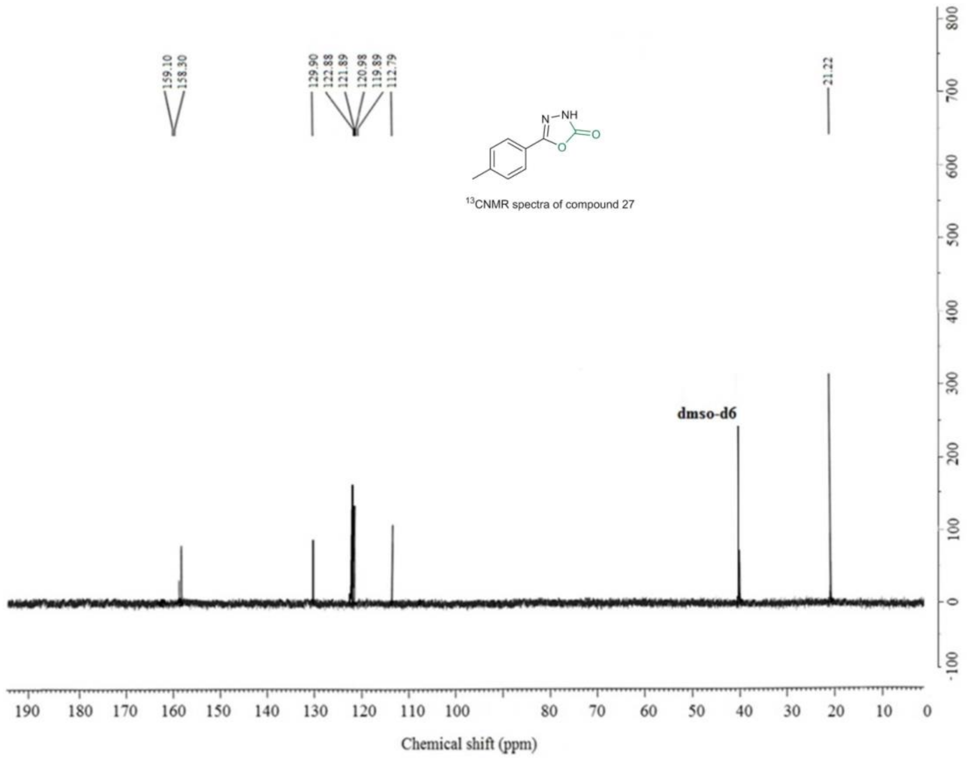

^{13}CNMR spectra of compound 27

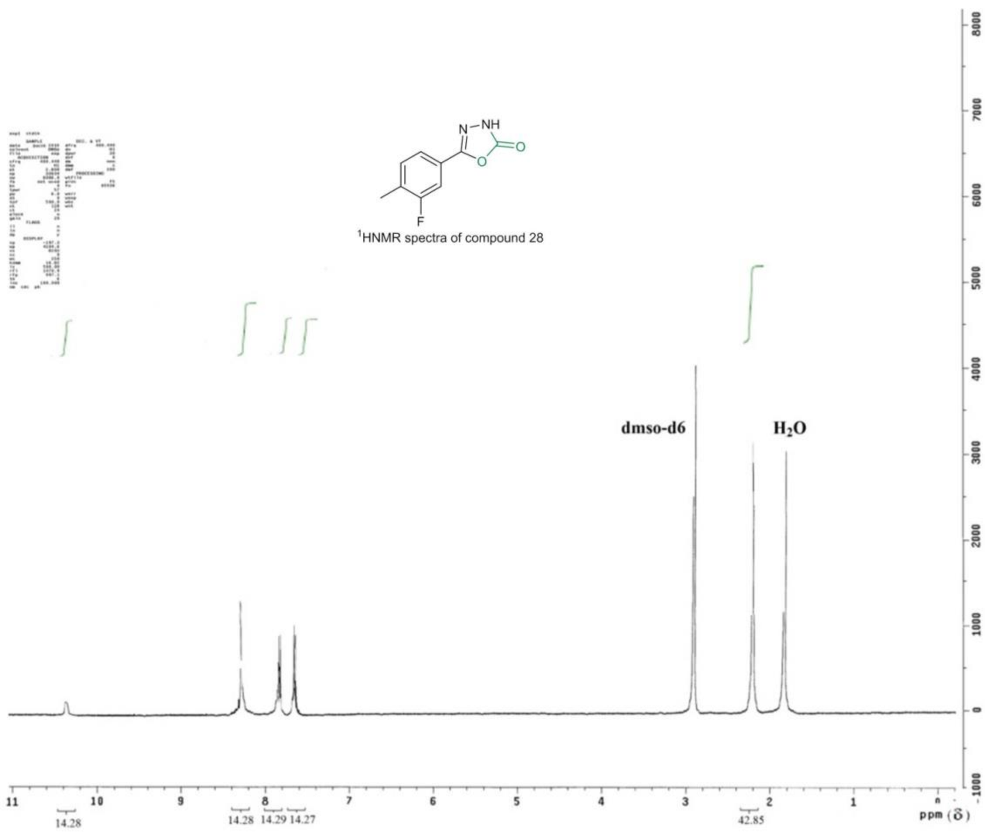
N–NH
O
F
^{1}HNMR spectra of compound 28
dmso-d6
H_2O
11 10 9 8 7 6 5 4 3 2 1 0
ppm (δ)
14.28
14.28 14.29 14.27
42.85
8000 7000 6000 5000 4000 3000 2000 1000 0 -1000

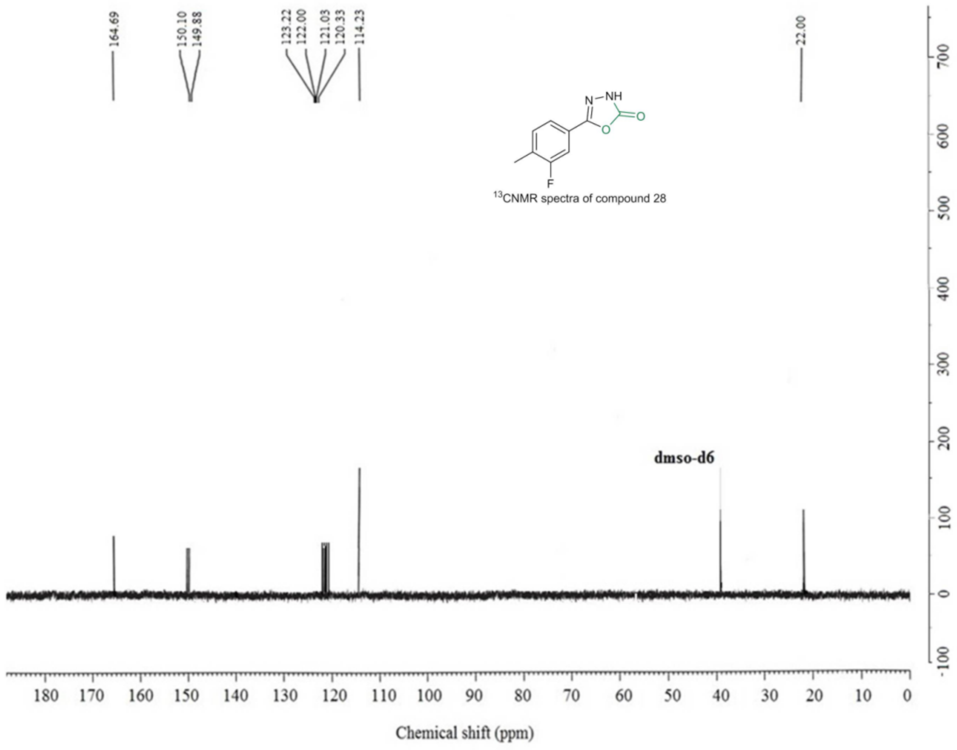
164.69
150.10
149.88
123.22
122.00
121.03
120.33
114.23
22.00
N–NH
O
F
^{13}CNMR spectra of compound 28
dmso-d6
180 170 160 150 140 130 120 110 100 90 80 70 60 50 40 30 20 10 0
Chemical shift (ppm)
700 600 500 400 300 200 100 0 -100

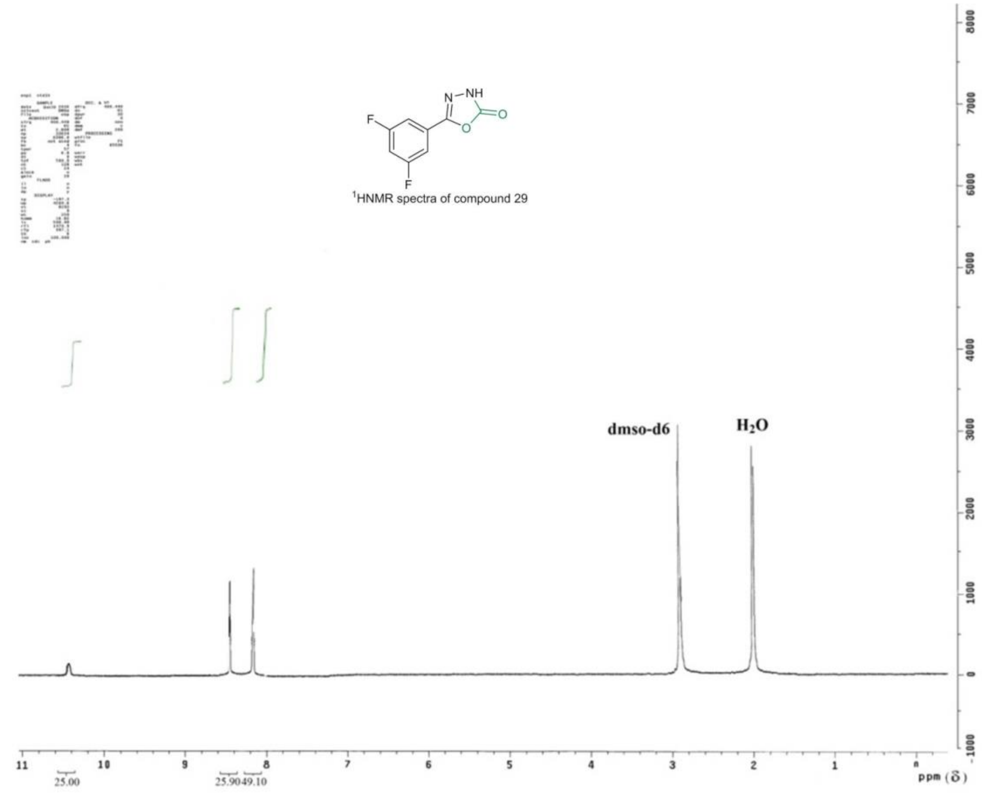

N–NH
F
O
O
F
¹HNMR spectra of compound 29
dmso-d6
H₂O
25.00
25.90
49.10
ppm (δ)

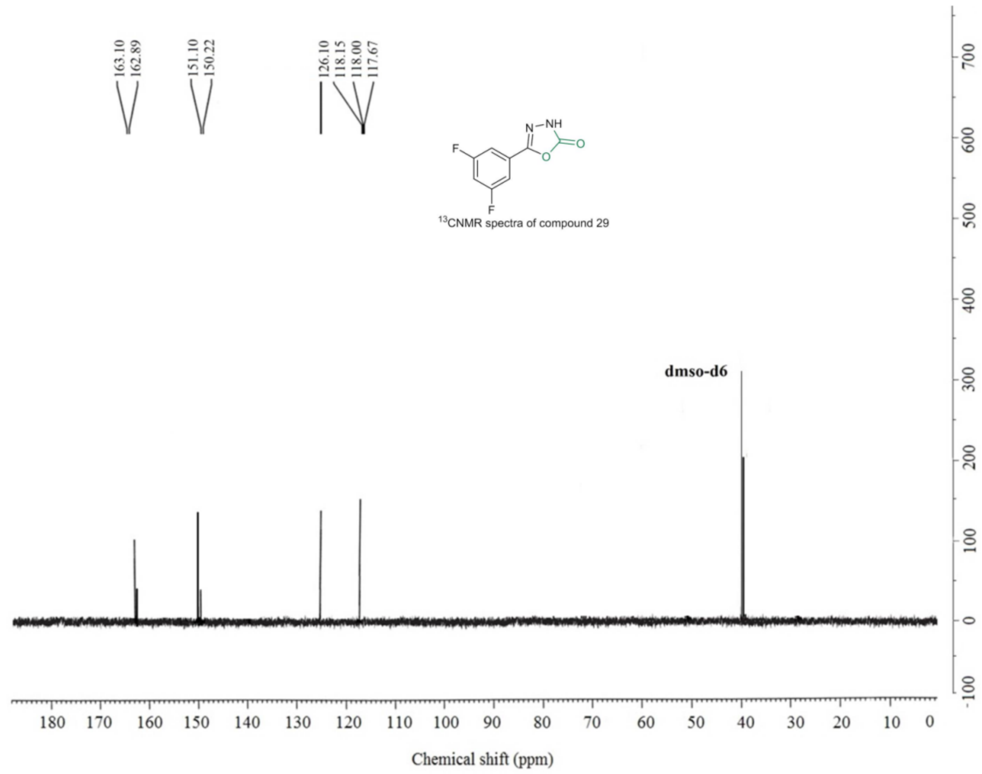

163.10
162.89
151.10
150.22
126.10
118.15
118.00
117.67
¹³CNMR spectra of compound 29
dmso-d6
Chemical shift (ppm)
